# RNA Synthesis and Capping by Non-segmented Negative Strand RNA Viral Polymerases: Lessons From a Prototypic Virus

**DOI:** 10.3389/fmicb.2019.01490

**Published:** 2019-07-10

**Authors:** Tomoaki Ogino, Todd J. Green

**Affiliations:** ^1^Department of Molecular Biology and Microbiology, Case Western Reserve University School of Medicine, Cleveland, OH, United States; ^2^Department of Inflammation and Immunity, Lerner Research Institute, Cleveland Clinic, Cleveland, OH, United States; ^3^Department of Microbiology, School of Medicine, University of Alabama at Birmingham, Birmingham, AL, United States

**Keywords:** non-segmented negative strand RNA viruses, vesicular stomatitis virus, rabies virus, transcription, replication, RNA-dependent RNA polymerase, mRNA capping, GDP polyribonucleotidyltransferase

## Abstract

Non-segmented negative strand (NNS) RNA viruses belonging to the order *Mononegavirales* are highly diversified eukaryotic viruses including significant human pathogens, such as rabies, measles, Nipah, and Ebola. Elucidation of their unique strategies to replicate in eukaryotic cells is crucial to aid in developing anti-NNS RNA viral agents. Over the past 40 years, vesicular stomatitis virus (VSV), closely related to rabies virus, has served as a paradigm to study the fundamental molecular mechanisms of transcription and replication of NNS RNA viruses. These studies provided insights into how NNS RNA viruses synthesize 5′-capped mRNAs using their RNA-dependent RNA polymerase L proteins equipped with an unconventional mRNA capping enzyme, namely GDP polyribonucleotidyltransferase (PRNTase), domain. PRNTase or PRNTase-like domains are evolutionally conserved among L proteins of all known NNS RNA viruses and their related viruses belonging to *Jingchuvirales*, a newly established order, in the class *Monjiviricetes*, suggesting that they may have evolved from a common ancestor that acquired the unique capping system to replicate in a primitive eukaryotic host. This article reviews what has been learned from biochemical and structural studies on the VSV RNA biosynthesis machinery, and then focuses on recent advances in our understanding of regulatory and catalytic roles of the PRNTase domain in RNA synthesis and capping.

## Introduction

The order *Mononegavirales* comprises highly diversified eukaryotic viruses with a monopartite negative strand RNA genome (rarely bipartite genomes), which includes important human pathogens [e.g., rabies virus (RABV), measles virus (MeV), Nipah virus (NiV), human respiratory syncytial virus (HRSV), Ebola virus (EBOV)] (Lamb, 2013). Since gene products as well as RNA genomes of these non-segmented negative strand (NNS) RNA viruses share structural and functional similarities, they are believed to have evolved from a common ancestor. The order *Mononegavirales* was originally established with three families, *Rhabdoviridae, Paramyxoviridae* (including two subfamilies, *Paramyxovirinae*, and *Pneumovirinae*), and *Filoviridae* (Pringle, 1991). Over the past two decades, the order *Mononegavirales* was expanded through discoveries of numerous new viruses into 11 families, including the classical three families, *Pneumoviridae* (elevated from the *Pneumovirinae* subfamily), *Bornaviridae, Nyamiviridae, Mymonaviridae, Sunviridae, Artoviridae, Lispiviridae*, and *Xinmoviridae* (Maes et al., 2019).

**FIGURE 1:**
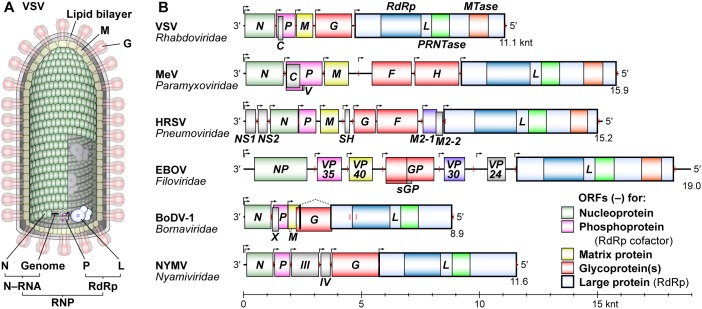
Schematic diagrams of a VSV virion and NNS RNA viral genomes. **(A)** A VSV particle is composed of a cellular lipid bilayer, viral RNA genome, and five viral proteins: nucleo- (N), phospho- (P), matrix (M), glyco- (G), and large (L) proteins. The virus particle contains a ribonucleoprotein (RNP) composed of N–RNA and RNA-dependent RNA polymerase (RdRp) complexes. **(B)** The gene organization of negative-strand genomes of typical NNS RNA viruses [measles virus (MeV), human respiratory syncytial virus (HRSV), Ebola virus (EBOV), Borna disease virus 1 (BoDV-1), and Nyamanini virus (NYMV)] belonging to different families is depicted in the 3′ to 5′ order. Transcription initiation and termination sites are shown by bent arrows and red vertical lines, respectively. The positions of negative-strand open reading frames are shown by colored boxes. The *L* genes encode an L protein with RdRp, GDP polyribonucleotidyltransferase (PRNTase), and methyltransferase (MTase, except for nuclear-replicating viruses) domains. A scale bar is shown at the bottom (knt, kilo nucleotides).

Vesicular stomatitis Indiana virus [hereafter simply called vesicular stomatitis virus (VSV)] is an arthropod-borne animal virus belonging to the *Vesiculovirus* genus in the *Rhabdoviridae* family. VSV has served as a prototype to elucidate the fundamental molecular mechanisms of transcription and replication of NNS RNA viruses, since its virion-associated RNA-dependent RNA polymerase (RdRp) activity was discovered (Baltimore et al., 1970). A bullet-shaped VSV particle contains a single-strand RNA genome of 11,161 nucleotides (nt), which is encapsidated with the nucleo- (N) proteins to form a helical nucleocapsid (called the N–RNA complex/template) (Green et al., 2006; Ge et al., 2010; Figure 1A). An RdRp complex is composed of the catalytic large (L) protein and its co-factor phospho- (P) protein (Emerson and Yu, 1975), and is associated with the N–RNA complex to assemble a ribonucleoprotein (RNP) complex (Mellon and Emerson, 1978). In the virus particle, the RNP complex is coated with a layer composed of the matrix (M) proteins (Ge et al., 2010), which is further wrapped by a lipid bilayer envelope studded with the glyco- (G) proteins (reviewed in Lyles et al., 2013).

The VSV genome consists of five structural genes, *N, P, M, G*, and *L*, which are arranged in tandem from the 3′- to 5′-end (Figure 1B). NNS RNA viruses belonging to the *Rhabdoviridae* (e.g., RABV), *Paramyxoviridae* (e.g., MeV), *Pneumoviridae* (e.g., HRSV), *Filoviridae* (e.g., EBOV), *Bornaviridae* [e.g., Borna disease virus-1 (BoDV-1)], *Nyamiviridae* [e.g., Nyamanini virus (NYMV)], and other families share the same gene organization with VSV, but have diversified their structural genes and often acquired additional structural and/or non-structural genes during evolution (Lamb, 2013; Amarasinghe et al., 2018). Despite vastly different primary structures of P proteins and their counterparts (e.g., EBOV VP35), these RdRp co-factors may play similar roles in transcription and replication [reviewed in Jamin and Yabukarski (2017)]. L proteins are the most conserved proteins among NNS RNA viruses, and catalyze all enzymatic reactions required for viral RNA synthesis and processing (Emerson and Yu, 1975; Hercyk et al., 1988; Hunt and Hutchinson, 1993; Sleat and Banerjee, 1993; Grdzelishvili et al., 2005; Li et al., 2005; Ogino et al., 2005; Ogino and Banerjee, 2007; Galloway and Wertz, 2008; Rahmeh et al., 2009; Morin et al., 2012; Paesen et al., 2015; Jordan et al., 2018). In this review article, we outline the lessons learned from five decades of biochemical and structural studies on NNS RNA viral replication using VSV as a prototype, and focus on recent findings regarding unique roles of rhabdoviral L proteins in RNA synthesis and cap formation.

## Transcription and Replication of the VSV Genome

Since VSV can be easily and safely isolated from cell culture supernatants and also displays the highest RdRp activity *in vitro* among known NNS RNA viruses [e.g., RABV, Sendai virus (SeV, *Paramyxoviridae*), Newcastle disease virus (*Paramyxoviridae*), MeV, HRSV], VSV has proven to be a remarkable model to elucidate the mechanisms of RNA biogenesis by NNS RNA viruses. Early studies using *in vitro* transcription systems with detergent disrupted VSV particles and purified RNPs revealed that VSV packages all enzymes required for primary transcription into virions, including RdRp (Baltimore et al., 1970), capping enzyme (Abraham et al., 1975a,b), MTases (Abraham et al., 1975b; Testa and Banerjee, 1977), and poly(A) polymerase (Banerjee and Rhodes, 1973; Villarreal and Holland, 1973; Banerjee et al., 1974) activities. *In vitro* reconstitution studies demonstrated that the N–RNA complex, rather than a naked RNA genome, serves a template for transcription and the L and P (previously called NS) proteins are catalytic and regulatory subunits, respectively, of the RdRp complex (Emerson and Wagner, 1972; Emerson and Yu, 1975; Naito and Ishihama, 1976; De and Banerjee, 1984, 1985).

**FIGURE 2:**
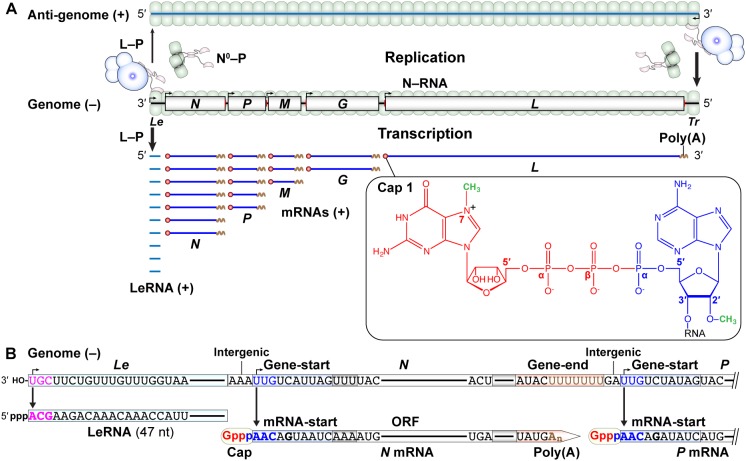
Transcription and replication of the VSV genome. **(A)** The negative-strand VSV genome in the N–RNA complex serves as a template for transcription (lower) and replication (upper). *Le* and *Tr* denote the terminal leader and trailer regions, respectively, in the genome. According to the single-entry, stop-start transcription model, the L–P RdRp complex enters from the 3′-end of the genome and sequentially synthesizes the leader RNA (LeRNA) and five monocistronic mRNAs with a 5′-cap 1 structure and 3′-poly(A) tail (lower). A GDP moiety (red) of GTP, an AMP moiety (blue) of ATP, and two methyl groups (green) are incorporated into the cap 1 structure. The L–P and N^0^–P (N^0^: RNA-free N) complexes are required for encapsidation-coupled genome replication (upper). **(B)** LeRNA is synthesized from the 3′-terminal of the *Le* promoter in the genome. The conserved gene-start and gene-end sequences serve as internal transcription initiation and termination/polyadenylation signals, respectively. The conserved 5′-terminal mRNA-start sequence acts as a signal for mRNA capping.

The negative-strand VSV genome begins and ends with the short 3′-leader (*Le*) and 5′-trailer (*Tr*) sequences, respectively, and contains the five internal genes that are tandemly connected via intergenic regions (Figure 2A,B). The genome serves as a template for synthesis of positive-strand mRNAs and an anti-genome, the latter of which is further copied into progeny genomes during replication. Using *in vitro* reconstituted N–RNA templates with synthetic oligo-RNAs and purified N proteins, the 3′-terminal UGC sequence in the VSV *Le* promoter was identified as the minimum promoter that is critical for terminal *de novo* initiation with a native L–P complex (Smallwood and Moyer, 1993). However, additional residues within the first 18 nt of the VSV genome are also necessary for efficient replication of a mini-replicon in cultured cells (Li and Pattnaik, 1999). Each gene begins with the gene-start sequence (3′-UUGUCDNUAG; D: A, U, or G; N: any nucleotide) and ends with the gene-end (3′-AUACUUUUUUU) sequence, which play critical roles in transcription initiation/capping and termination/polyadenylation, respectively (Abraham et al., 1975a,b; Schubert et al., 1980; Iverson and Rose, 1981; Schnell et al., 1996; Barr et al., 1997; Stillman and Whitt, 1999; Barr and Wertz, 2001; Figure 2B).

According to the “single-entry, stop-start transcription” model suggested from *in vitro* studies (Testa et al., 1980; Emerson, 1982), the VSV RdRp enters from the 3′ end of the *Le* promoter in the genome to initiate synthesis of the leader RNA (LeRNA) of ∼47 nt (Colonno and Banerjee, 1976, 1978; Testa et al., 1980). After synthesis of LeRNA, the VSV RdRp reinitiates transcription at the *N* gene-start sequence to generate *N* mRNA with a 5′-terminal cap structure [cap 1, m^7^G(5′)ppp(5′)Am-: *N*^7^-methylguanosine(5′)triphospho(5′)2′-*O*-methyladenosine] (Abraham et al., 1975b). The 3′-UYG (Y: U or C) sequence in the gene-start sequence and its complementary sequence (5′-ARC; R: A or G) in the conserved mRNA start-sequence (5′-AACAGHNAUC; H: U, C, or A) are essential for transcription initiation (Stillman and Whitt, 1999) and mRNA capping (Ogino and Banerjee, 2007, 2008), respectively. The VSV RdRp adds a poly(A) tail (∼200 nt) to the 3′-end of *N* mRNA by slippage at the U7 tract in the gene-end sequence (Schubert et al., 1980; Iverson and Rose, 1981; Barr et al., 1997). After releasing *N* mRNA, the same RdRp similarly initiates and terminates transcription at gene-start and gene-end sequences, respectively, for each gene to sequentially synthesize *P, M, G*, and *L* mRNAs (Abraham and Banerjee, 1976; Ball and White, 1976; Testa et al., 1980; Iverson and Rose, 1981). Reducing the efficiency to ∼70% in transcription reinitiation at each gene junction results in the formation of a gradient in transcript abundance in the following order: *Le* > *N* > *P* > *M* > *G* > *L* (Iverson and Rose, 1981). Alternatively, it has also been proposed that the sequential mRNA synthesis occurs independently of LeRNA synthesis in VSV-infected cells (Whelan and Wertz, 2002). However, it is still not known how the VSV RdRp reaches the internal *N* gene-start sequence by bypassing the 3′-terminal *Le* sequence of the genome *in cellula*.

To replicate the VSV genome, the VSV RdRp needs to switch its mode from transcription to replication. During replication, the VSV RdRp ignores the termination signal at the end of the *Le* region, and throughout the genome to generate the full-length antigenome, which should be co-replicationally encapsidated with the N proteins. Selective encapsidation of LeRNA with the N protein may trigger switching from transcription to replication coupled with nucleocapsid assembly (Blumberg et al., 1981, 1983). Complexes between the RNA-free N (N^0^) and P proteins (called N^0^–P) accumulated in infected cells play a key role in co-replicational encapsidation of the antigenome or genome (Peluso, 1988; Peluso and Moyer, 1988). The N^0^–P complex was partially purified from infected cells as a soluble viral factor required for encapsidation-dependent replication of the VSV genome as well as its defective-interfering particle genome (Peluso, 1988; Peluso and Moyer, 1988). The P protein was suggested to prevent aggregation and non-specific RNA binding of the N protein by forming the N^0^–P complex (Masters and Banerjee, 1988). Furthermore, a recombinant form of the N^0^–P complex was shown to inhibit transcription and to promote replication of the VSV genome in the presence of cell extracts, indicating that the N^0^–P complex serves as a *bona fide* switching factor from transcription to replication (Gupta and Banerjee, 1997). Thus, the P protein acts as a chaperone for the N protein to specifically encapsidate newly synthesized antigenome or genome. Furthermore, an L–P–N tripartite complex, rather than the L–P complex, efficiently performs replication in the presence of the N^0^–P complex (Gupta et al., 2003), suggesting that the tripartite complex may act as a replicase.

## The N Protein

The VSV N protein (422 amino acids) is a capsid protomer for the intact nucleocapsid, the assembled N/RNA polymer (Green et al., 2014). Studies to determine the structure of the VSV virions and nucleocapsids began in the 1960s using negative stain electron microscopy (Howatson and Whitmore, 1962; McCombs et al., 1966; Simpson and Hauser, 1966; Nakai and Howatson, 1968). These early studies noted the virion morphology, along with size and the repeating nature of the nucleocapsid. Prior to being packaged into the virion and perhaps while serving as the active template for transcription and replication, the nucleocapsid exists in multiple morphological states in the cell, including: an undulating ribbon, a loosely coiled helix, and a tightly coiled helix that is usually found at the termini of the nucleocapsids (Howatson and Whitmore, 1962; McCombs et al., 1966; Simpson and Hauser, 1966; Nakai and Howatson, 1968; Thomas et al., 1985; Desfosses et al., 2013). Helical states of the nucleocapsid are a common characteristic of the nucleocapsids of the NS RNA viruses as members of the paramyxoviruses (Egelman et al., 1989) and orthomyxoviruses (Heggeness et al., 1982) also exist in varied helical states. Ultimately, the nucleocapsid has the structural role of forming the internal scaffold of the intact virion (Ge et al., 2010; Desfosses et al., 2013). The structure of the intact virion determined by cryo-EM reconstruction methods showed placement and organization of the lipid bilayer, and the N and M proteins (Ge et al., 2010) and later the G protein (Si et al., 2018). The nucleocapsid winds up to form the bullet shape, a morphology that can be driven by pH and ionic strength conditions in absence of other viral proteins (Desfosses et al., 2013). The M protein forms a chainmail layer around the nucleocapsid, stabilizing the bullet shape of the capsid in the virion, while the trimeric G is embedded in the viral membrane.

**FIGURE 3:**
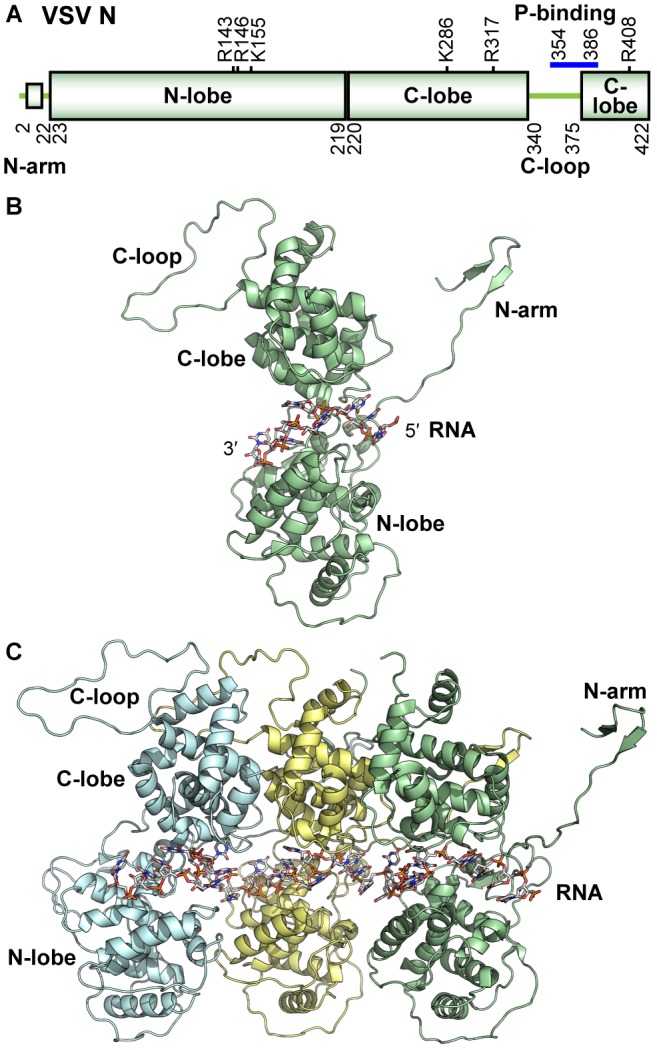
Structure of the VSV N protein. **(A)** The domain organization of the VSV N protein is schematically represented. Basic residues contributing to RNA binding are noted above the schematic, and residues involved in P-binding are noted with a blue bar. **(B)** A cartoon representation of the monomeric N protein (PDB id: 2GIC) is shown with bound nine-mer of RNA encapsidated and regional landmarks noted. **(C)** Assembled trimer of N proteins (each represented in a different color) with bound 27-mer of RNA is shown. All illustrations were prepared with PyMOL (DeLano, 2002).

Medium resolution reconstructions from electron microscopy studies of the VSV (Chen et al., 2004) and RABV (Schoehn et al., 2001) N proteins assembled into nucleocapsid-like particles (assembled N protein with encapsidated RNA) from recombinant sources (Green et al., 2000; Schoehn et al., 2001) showed that the N protein had a bi-lobed structure. The high-resolution structure of VSV (Green et al., 2006) and RABV (Albertini et al., 2006) nucleocapsid-like particles were determined by X-ray crystallography. The crystal structures confirmed that the N protein contains an N-terminal (N-lobe) and a C-terminal (C-lobe) lobe (Figure 3A,B). These lobes were comprised almost exclusively of α helices and have since been shown to have a common topology for the NS RNA virus N proteins (Green et al., 2014). Two main projections extend from these lobes: a 22-amino acid (N-terminal arm) that precedes the N-lobe and an extended loop projects from within the C-lobe (C-loop, residues 340–375). The N-arm and C-loop are critical for the formation of the assembled nucleocapsid (Zhang et al., 2008), which is created by the polymerization of the N protein concomitant with RNA encapsidation at the site of replication. In the nucleocapsid structure, each monomer of the N protein makes cross-molecular contacts with three neighboring N subunits (Figure 3C, 4B). These contacts include: the interactions between (I) the N-arm and the C-lobe on the proximal surface of the left neighboring subunit, (II) the C-loop and the C-lobe of the neighboring subunit to the right, and (III) the N-arm and the C-loop of the N protein subunit two units away on the left. Each of these unique interactions is repeated to generate the assembled nucleocapsid. Weakening the interactions between adjacent N protein molecules in the nucleocapsid has been shown to alter the levels of RNA synthesis directed from RNP templates (Harouaka and Wertz, 2009). The extreme C-terminus of N is also important for RNA encapsidation (Heinrich et al., 2012). Thus structural features on N are key to regulating N protein function.

**FIGURE 4:**
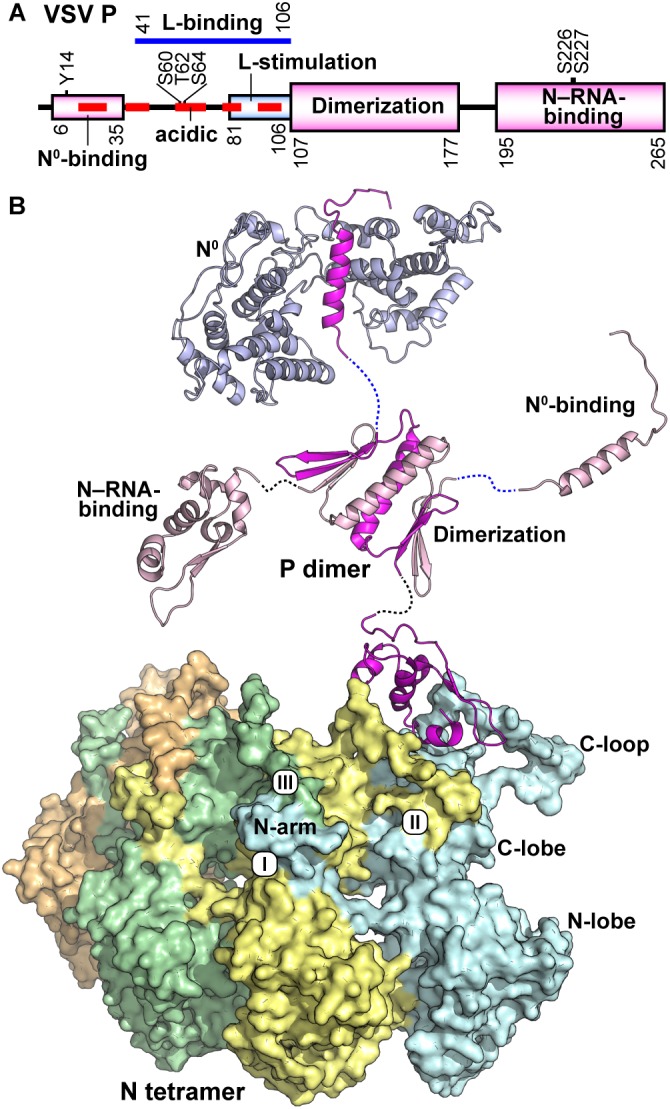
Structure of the VSV P protein. **(A)** The domain organization of the VSV P protein is schematically represented. Domains are labeled according to known binding partners and function. Six phosphorylation sites are noted above the schematic. **(B)** VSV P exists as a dimer and is represented in cartoon form with regional aspects noted in **(A)** labeled. P binds the unassembled N^0^ (top) via a single helix and adjacent amino acids in the N-terminal intrinsically disordered region (PDB id: 3PMK). The dimerization domain (PDB id: 2FQM) is shown central to the figure. The C-terminal domain of P (PDB id: 3HHZ) binds a bipartite binding site involving the C-loops and an α-helix in the C-lobe of N. A tetramer of N proteins (each represented in a different color) is shown in surface representation. The view is 180 degrees from that in Figure 3C. The three contacts that generate the nucleocapsid are noted: the interactions between (I) the N-arm and the C-lobe on the proximal surface of the left neighboring subunit, (II) the C-loop and the C-lobe of the neighboring subunit to the right, and (III) the N-arm and the C-loop of the N protein subunit two units away on the left.

The VSV and RABV N protein structures also revealed detail on how RNA is stored by the capsid as well as the unique structure adopted by the RNA upon encapsidation. When the N protein subunits assemble to form the nucleocapsid, they generate a continuous tunnel that sequesters the RNA upon encapsidation and protecting the RNA against degradation by nuclease and base-catalyzed hydrolysis (Keene et al., 1981; Emerson, 1987; Green et al., 2000; Iseni et al., 2000). The N- and C-lobes angle together to form a cavity, where each N protein monomer encapsidates nine ribonucleotides (Figure 3B). The RNA forms two-quasi helical motif structures. In motif one, bases one to four (counting from 5′ to 3′) are stacked to resemble a single strand of a type-A helical RNA (half of the typical RNA duplex) and face away from the protein cavity. In motif two, bases of nucleotides five, seven, and eight are then stacked while facing the interior of the protein cavity, while nucleotide six is rotated opposite of these three bases to alone face the solvent side of the cavity. Nucleotide nine is located between neighboring N subunits and is transitioned between the two helical RNA structures sometimes in line with the base of nucleotide one, when adenine or cytosine are in this position (Green et al., 2011). This unique helical RNA pattern is repeated throughout the nucleocapsid (Albertini et al., 2006; Green et al., 2006; Ge et al., 2010). In the cavity, both lobes of the N protein contribute many positively charged and polar residues that interact with the negatively charged RNA backbone. Residues making hydrogen bond contacts with the nucleotides in RNA helix motif two were indispensable to the encapsidation of RNA and the production of templates that can support RNA synthesis, though residues shown to bind to motif one were not (Rainsford et al., 2010). RNA sequence specific interactions have also been observed (Green et al., 2011). Structures of nucleocapsid-like particles with encapsidated homopolymeric sequences of RNA revealed that each sequence had differing tightness of RNA stacking and patterns of interaction with the N protein. The relevance of these observations is currently unclear though genomic sequence motifs play many roles in both transcription and replication, as noted above.

The N and P proteins are intimately associated at different stages of the viral replication cycle. These associations require different modes of binding, using different regions of each protein. For the N^0^–P complex, the N-terminal helical region of P (residues 17–31) interacts with the central hinge region of N (Figure 4B), adjacent to the RNA binding cavity (Leyrat et al., 2011a). P bound in this state competes with both RNA binding and N polymerization. Post capsid-assembly and during transcription and replication, the P protein must interact with the nucleocapsid to deliver the L protein to the RNA template (Mellon and Emerson, 1978; Ogino et al., 2019). In this case, the C-terminal domain of the P protein binds to two neighboring N protein subunits within the nucleocapsid (Figure 4B; Green and Luo, 2009). The C-lobes of these adjacent N monomers form a unique bipartite binding site to accommodate P. The binding site of N includes α helix 13 of one subunit and the C-loops of both. This site is adjacent to the C-loop, the binding surface that receives the N-arm as well as the point of interaction of the N-arm/C-loop directly. Some local conformational changes in the capsid are necessary for the polymerase to gain access to the genomic RNA during transcription and replication. P binding to this site on the nucleocapsid has been suggested to destabilize the association of these critical elements potentially leading to the necessary conformational changes. Mutational analysis of N near the P binding site has shown that some residues in this region are crucial to viral transcription and/or replication (Harouaka and Wertz, 2009).

## The P Protein

The VSV P protein (265 amino acids) is a dimeric, non-globular molecule containing structured domains and disordered regions (Ding et al., 2006; Gerard et al., 2007; Green and Luo, 2009; Jamin and Yabukarski, 2017; Figure 4A,B). During transcription and replication of the VSV genome, the P protein carries out multiple functions, such as bridging the L protein and the N–RNA template (Mellon and Emerson, 1978), stimulating RNA synthesis with the L protein at both the initiation and elongation steps (Emerson and Yu, 1975; De and Banerjee, 1984, 1985; Williams et al., 1988; Morin et al., 2012; Ogino et al., 2019), and chaperoning the N^0^ protein to the replication complex (Masters and Banerjee, 1988; Peluso, 1988; Peluso and Moyer, 1988; Gupta and Banerjee, 1997). The N-terminal half of the P protein contains regions required for interactions with the RNA-free N^0^ protein (residues 6–35) (Chen et al., 2007; Leyrat et al., 2011a) and the L protein [a region(s) within residues 41–106] (Emerson and Schubert, 1987; Chen et al., 2007; Rahmeh et al., 2012), while its C-terminal region (residues 195–265) is involved in binding to the N–RNA template (Das et al., 1997; Green and Luo, 2009). The dimerization domain (including residues 107–177) resides centrally in the P protein (Chen et al., 2006; Ding et al., 2006).

X-ray crystallographic (Leyrat et al., 2011a), NMR, and SAXS (Leyrat et al., 2011b, 2012) studies have shown that the N-terminus of P is largely intrinsically disordered with only residues 17–31 having an ordered secondary structure, a single α helix that binds to N^0^ (Figure 4B; Leyrat et al., 2011a). VSV P exists as a functional dimer and dimerization is achieved by a unique domain-swapping arrangement by amino acid residues 112–169 (Ding et al., 2006). The fold of this domain consists of two β hairpins separated by an internal α helix. The α helix from each monomer, together form the centerpiece of the dimer, while β hairpin one interacts with β hairpin two of the opposite monomer to form a four-stranded β sheet on each side of the assembly (Figure 4B). The C-terminal region of VSV P (amino acid residues 195–265) forms a single monomeric domain (Ribeiro, Jr., Favier et al., 2008; Green and Luo, 2009) containing a four-α helical bundle that is flanked on one side by a single β hairpin (Figure 4B). This topology is maintained upon binding to the assembled nucleocapsid (Green and Luo, 2009).

The VSV P protein is known to be phosphorylated at S60, T62, and S64 in the N-terminal highly acidic region (Barik and Banerjee, 1992a; Das et al., 1995; Gupta et al., 1995), at S226 and S227 in the C-terminal N-RNA binding domain (Barik and Banerjee, 1992b; Chen et al., 1997), and at Y14 in the N^0^-binding domain (Mondal et al., 2014). Previous studies suggested that phosphorylation of P modulates P-oligomerization (Gao and Lenard, 1995b), L–P complex formation (Das et al., 1995; Gao and Lenard, 1995a,b; Spadafora et al., 1996), and transcription/replication (Barik and Banerjee, 1992a,b; Pattnaik et al., 1997; Hwang et al., 1999; Mondal et al., 2014).

The L-stimulatory domain (residues 81–106) (Rahmeh et al., 2012) resides within the N-terminal L-binding region and increases the specific RdRp activity of the L protein at both steps of RNA synthesis, namely *de novo* initiation and elongation (Morin et al., 2012; Rahmeh et al., 2012; Ogino et al., 2019). In addition to the L-stimulatory domain, the C-terminal N–RNA-binding domain (Green and Luo, 2009) is required for terminal *de novo* initiation from the N–RNA template, but not from a naked 20-nt oligo-RNA template with the VSV *Le* promoter, to catalyze the first phosphodiester bond formation (AC synthesis) (Ogino et al., 2019). Although a low concentration (25 nM) of the recombinant VSV L protein can initiate transcription in the presence of the P protein from the naked RNA template, a 500–1000-fold higher concentration of the naked RNA template is required than that of the N–RNA template for efficient initiation (Ogino et al., 2019). Thus, the interaction between the C-terminal domain of the P protein and the C-terminal lobe of two adjacent N proteins located at the 3′-end of the genome (Green and Luo, 2009) appears to dramatically enhance template recognition with the L protein to carry out terminal *de novo* initiation from the N–RNA template. On the other hand, a high concentration (0.2 μM) of recombinant VSV L protein was reported to initiate transcription from a naked RNA template with a 19-nt VSV *Le* promoter sequence (0.2 μM) in the absence of the P protein (Morin et al., 2012), although its specific activity is not reported, suggesting that the high concentrations of the L protein and template may tolerate the absence of the P protein. Under these *in vitro* conditions, the P protein stimulates overall transcription three- to fourfold and the processivity of the L protein, which tends to terminate transcription prematurely in the absence of the P protein (Morin et al., 2012).

## The L Protein

**FIGURE 5:**
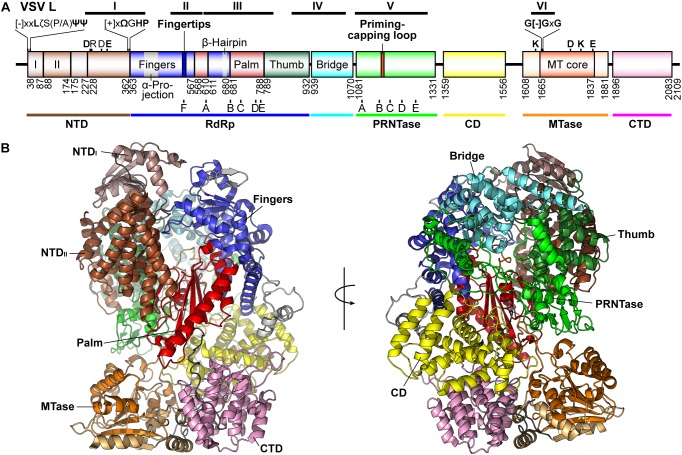
Structure of the VSV L protein. **(A)** The domain organization of the VSV L protein is schematically represented. Proposed domains and subdomains are colored differently. Numbers denote the positions of the amino acid residues starting and ending respective domains/subdomains. The N-terminal domain (NTD) is composed of two subdomains (I, light brown; II, brown). The RNA-dependent RNA polymerase (RdRp) domain contains the fingers (blue), palm (red), and thumb (dark green) subdomains. The RdRp domain is connected to the GDP polyribonucleotidyltransferase (PRNTase) domain through a bridge domain (cyan), which may have a similar role to the bridge domain of the La Crosse orthobunyavirus (LACV) L protein (Gerlach et al., 2015). A C-terminal region consists of a connector domain (CD, yellow), methyltransferase (MTase, light orange) domain with a SAM-dependent MTase core fold (orange), and C-terminal domain (CTD, pink). The positions of RdRp (A–F) and PRNTase (A–E) motifs are indicated below respective domains. Other known motifs and structural elements are indicated above the diagram. The positions of the originally reported six conserved regions (CR or blocks I–VI) are shown on the top. [-], [+], ζ, Ψ, Ω, and x indicate negatively charged, positively charged, hydrophilic, aliphatic, aromatic, and any amino acids, respectively. **(B)** Two views of the three-dimensional structure of the VSV L protein (PDB id: 5A22) are represented as ribbon models. The domains and subdomains are colored as in **(A)**.

Non-segmented negative strand RNA viral L proteins share six conserved regions (called CRs or blocks I–VI) (Poch et al., 1990), in which blocks III and VI were predicted to be parts of RdRp (Poch et al., 1990) and MTase (Bujnicki and Rychlewski, 2002; Ferron et al., 2002) domains, respectively (Figure 5A). Consistently, conserved amino acid residues in the RdRp and MTase domains have been shown to be required for transcription (Sleat and Banerjee, 1993; Schnell and Conzelmann, 1995; Malur et al., 2002) and cap methylation (Grdzelishvili et al., 2005; Li et al., 2005, 2006), respectively. Although the precise P-binding sites in rhabdoviral L proteins are still unknown, a C-terminal part of the RABV L protein was reported to contain a P-binding site (Chenik et al., 1998; Castel et al., 2009; Nakagawa et al., 2017). In contrast, N-terminal parts of L proteins of paramyxoviruses (Parks, 1994; Chandrika et al., 1995; Holmes and Moyer, 2002) and filoviruses (Becker et al., 1998; Trunschke et al., 2013) are required for binding to their cognate P and VP35, respectively.

The first electron microscopic (EM) analysis of the VSV L protein (2,109 amino acids) revealed that it consists of an N-terminal ring-like structure and a flexible C-terminal appendage containing three globular domains, which were predicted to be responsible for RNA synthesis and cap formation, respectively (Rahmeh et al., 2010). The P protein as well as its N-terminal fragment was found to induce large conformational changes in the L protein to form a rigid structure (Rahmeh et al., 2010, 2012). A recent high-resolution structural analysis of the VSV L protein complexed with a fragment of the P protein (residues 35–106) at 3.8 Å by cryo-EM showed that the N-terminal ring-like structure is composed of the RdRp domain containing blocks I to III (residues 35–866) and capping domain with blocks IV and V (called Cap, residues 866–1334), and the C-terminal three globular domains correspond to the connecter (CD, residues 1358–1557), methyltransferase (MT, residues 1598–1892), and C-terminal (CTD, residues 1893–2092) domains (Liang et al., 2015; Figure 5A). Here, based on structural similarities of the N-terminal and core RdRp domains of the VSV L protein to those of segmented negative strand RNA viruses [influenza viruses, La Crosse orthobunyavirus (LACV)] (Pflug et al., 2014; Reich et al., 2014; Gerlach et al., 2015; Hengrung et al., 2015), the N-terminal ring-like structure of the VSV L protein is reconsidered to be divided into plausible domains or subdomains (Figure 5A,B).

X-ray crystallographic analysis of the N-terminal region (residues 37–379) of the VSV L protein at 1.8 Å resolution showed that residues 43–371 constitute a flat domain (N-terminal domain, NTD) composed of two subdomains (I and II) (Qiu et al., 2016). The NTD shares topological similarity with the C-terminal domain of the influenza virus RdRp PA subunit (PA_C_) (He et al., 2008; Obayashi et al., 2008; Pflug et al., 2014; Reich et al., 2014; Hengrung et al., 2015), the PA_C_-like domain of the LACV RdRp L (Gerlach et al., 2015), and the N-terminal domain of the reovirus RdRp λ3 (Tao et al., 2002), suggesting that this evolutionally conserved domain decorating the RdRp core domain may have a common role in transcription among negative-strand and double-strand RNA viruses. Loop structures extended from the N- and C-termini of the VSV NTD contain conserved motifs, [-]xxLζS(P/A)ΨΨ ([-], negatively charged; ζ, hydrophilic; Ψ, aliphatic; x, any amino acid; VSV, 38-DYNLNSPLI-46) and [+]xΩGHP ([+], positively charged; Ω, aromatic amino acid; VSV, 356-RHWGHP-361), respectively, and are located in close proximity on the same molecular surface in the crystal structure of the NTD (Qiu et al., 2016). Some conserved and semi-conserved amino acid residues in these motifs (Y39, L41, L45, I46, R356, W358, H360, and P361) and in the NTD subdomain II (D236A, E290) of the VSV L protein are critical for RNA synthesis, but not for capping (Qiu et al., 2016). In our model of the VSV terminal initiation complex (Model Archive ^1^, id: ma-5k432) (Ogino et al., 2019), D290 and R356 are located very close to the triphosphate group of an initiator ATP.

**FIGURE 6:**
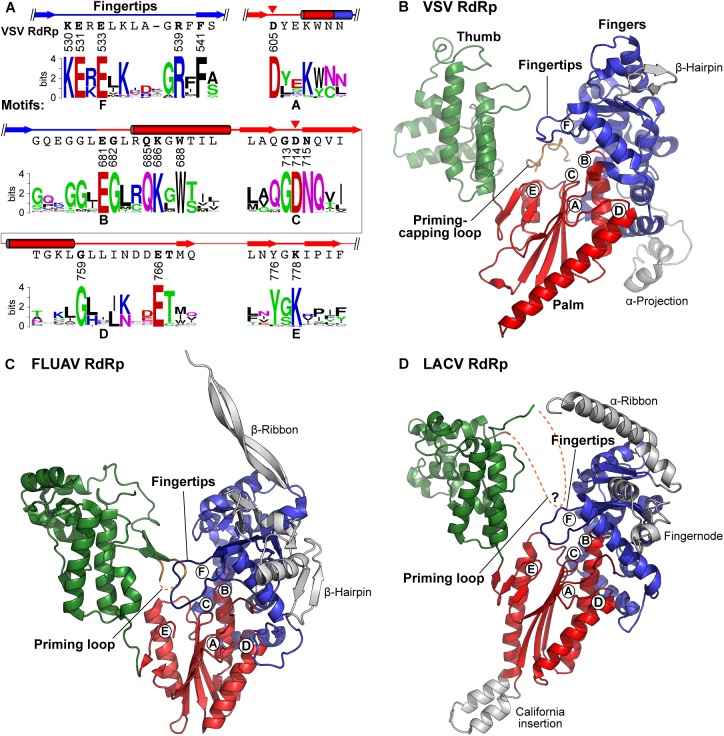
Structures of negative strand RNA viral RdRp domains. **(A)** Partial amino acid sequences containing RdRp motifs A–F of the VSV L protein are shown with their secondary structures (cylinders, α-helices; arrows, β-strands). The catalytic aspartate residues are indicated by red arrowheads. Amino acid sequence logos for RdRp motifs A–F in L proteins of 231 NNS RNA viruses belonging to the *Rhabdoviridae, Paramyxoviridae, Filoviridae, Bornaviridae*, and *Nyamiviridae* families (Maes et al., 2019) were generated using the WebLogo program (Crooks et al., 2004)^2^ as described in Neubauer et al. (2016). **(B–D)** The three-dimensional structural model of the RdRp domain of VSV **(B)** is compared with those of influenza A virus [FLUAV, PDB id: 4WSB (Pflug et al., 2014)] **(C)**, and LACV [PDB id: 5AMQ (Gerlach et al., 2015)] **(D)**. Their fingers, palm, and thumb subdomains are colored as in Figure 5A. The positions of RdRp motifs A–F are indicated by circled letters. The priming-capping loop of the VSV PRNTase domain and priming loops extended from the C-terminal regions of the FLUAV and LACV thumb subdomains are shown in orange. Missing regions of the loops in the structures are denoted by orange dashed lines. Other virus-specific substructures in their fingers subdomains are colored gray.

Similar to other viral RdRps (Poch et al., 1989; O’Reilly and Kao, 1998; Bruenn, 2003; Lang et al., 2013; te Velthuis, 2014; Reguera et al., 2016; Ferrero et al., 2018), the VSV RdRp domain is composed of fingers, palm, and thumb subdomains (Liang et al., 2015), and contains structural motifs A–F (Figure 6A,B). As proposed for all DNA and RNA polymerases (Steitz, 1998), two conserved aspartate residues, D605 and D714, in motifs A and C, respectively, of the VSV L protein can be predicted to serve as divalent metal ion-coordinating sites, where two metal ions bring the triphosphate group of an incoming NTP to the 3′-OH group of an initiator nucleotide or elongating transcript in close proximity. An activated 3′-oxyanion generated by deprotonation of the 3′-OH group nucleophilically attacks the α-phosphorus in the incoming NTP, resulting in the formation of a phosphodiester bond with concomitant release of inorganic pyrophosphate (PP_i_). Although there is a possibility that amino acid residues in the RdRp domain serve as a general base (e.g., D714 in motif C) and a general acid (e.g., R539 in motif F, K778 in motif E) to mediate deprotonation of the 3′-OH group and protonation of leaving PP_i_, respectively, as proposed for other polymerases (Florian et al., 2003, 2005; Castro et al., 2007, 2009; Gong and Peersen, 2010), two-metal-dependent nucleotide polymerization may proceed alternatively by a recently proposed self-activated mechanism involving direct proton transfer from the 3′-OH group of the incoming NTP to a PP_i_ group leaving from itself (Genna et al., 2016).

Motif B in a helix-turn motif leads from the fingers and forms part of the palm subdomain, and is involved in NTP-ribose selection, template binding, and RNA translocation (Gohara et al., 2000; Tao et al., 2002; Gong and Peersen, 2010; Garriga et al., 2013). In the modeled VSV terminal initiation complex (Ogino et al., 2019), the side-chain carbonyl group of Q685 in motif B is hydrogen-bonded to the 2′- and 3′-OH groups of the incoming CTP, and E681 interacts with K530 in motif F through a salt bridge. Motifs D and E form a strand-turn-helix and following β-hairpin structure, respectively, and may serve as scaffolds to build the palm subdomain. In some positive-strand RNA viruses, a lysine residue in motif D was suggested to act as a general acid to protonate the leaving PP_i_ (Castro et al., 2009) and to be important for the fidelity of nucleotide incorporation (Yang et al., 2012), though NNS RNA viral RdRp domains do not have any conserved basic amino acid residues in motif D. Motif E may be involved in positioning the 3′-end of elongating transcripts (Jacobo-Molina et al., 1993; Ferrer-Orta et al., 2007). In elongation complexes of the foot-and-mouth disease virus (picornavirus, positive-strand RNA virus) RdRp, basic amino acid residues in a motif E loop structure are associated with 3′-terminal residues of a primer RNA via hydrogen-bonding to its sugar-phosphate backbone (Ferrer-Orta et al., 2007). In the model of the VSV terminal initiation complex (Ogino et al., 2019), K778 in motif E is located in the vicinity of the triphosphate group of the initiator ATP (see Figure 10B), suggesting its important role in transcription initiation. Motif F in the “fingertips” structure contains conserved charged amino acids and is extended from the fingers subdomain toward the RdRp active site in the palm subdomain. Basic amino acids in motif F interact with the triphosphate group of the incoming NTP (Butcher et al., 2001; Bressanelli et al., 2002; Tao et al., 2002; Choi et al., 2004; Ferrer-Orta et al., 2004; Gong and Peersen, 2010; Appleby et al., 2015). Mutations of motif F in the SeV L protein abrogate transcription and replication without affecting P-binding (Smallwood et al., 1999). In the model of the VSV terminal initiation complex (Ogino et al., 2019), E533 and R539 in motif F are associated with the *C*^4^-amino group and α-phosphate, respectively, of the incoming CTP, and the aromatic side chain of F541 sits stacked in-line with 3′-nucleotides, U_1_ and G_2_, in a model template (3′-U_1_G_2_C_3_U_4_, see Figure 10B).

Similar to the RdRp domains in the influenza A virus PB1 [PDB id: 4WSB (Pflug et al., 2014)] (Figure 6C) and LACV L [PDB id: 5AMQ (Gerlach et al., 2015)] (Figure 6D) as well as other primer-independent RdRps (Lesburg et al., 1999; Butcher et al., 2001; Choi et al., 2004), we propose that the VSV RdRp domain (Figure 6B) has a large α-helical thumb domain (residues 789–932, 6 helices), which is larger than originally proposed (3 helices) (Liang et al., 2015). The extended region includes a part of block IV and contains a motif, GGx(11,12)Rx(3)D, which is located in a turn structure between two helices and conserved among NNS RNA viral RdRp domains. The thumb subdomain is connected to an mRNA capping enzyme domain (GDP polyribonucleotidyltransferase, PRNTase) domain via a functionally unknown α-helical subdomain (here called “bridge,” residues 939–1070, 7 helices) (Figure 5A,B), which does not have any highly conserved amino acid residues and may have a structural role similar to those of the bridge and lid domains of the LACV L protein closing its ring-like RdRp architecture (Gerlach et al., 2015). Based on the structure of the PRNTase domain obtained at a moderate resolution by cryo-EM, it was shown to have a flattened arrangement and appears to be formed on scaffolds provided from the thumb and bridge subdomains (see Figure 8). Although it seems more appropriate to refer to this region as a subdomain rather than a structurally separable domain, we designate it the PRNTase domain because it has originated as a functionally independent region of the NNS RNA viral L proteins.

The MTase domain of the VSV L protein is located between two functionally unknown domains, CD and CTD, and includes a typical *S*-adenosyl-L-methionine (SAM)-dependent MTase core fold with a glycine-rich SAM binding motif G[-]GxG (VSV, 1670-GDGSG-1674) and a 2′-*O*-MTase motif, namely K–D–K–E catalytic tetrad (VSV, K1651–D1762–K1795–E1833) (Bujnicki and Rychlewski, 2002; Ferron et al., 2002; Liang et al., 2015). Similar to the MTase domain of flaviviral NS5 proteins (Ray et al., 2006), the single MTase domain of NNS RNA viral L proteins was suggested to catalyze cap methylation at guanine (G)-*N*^7^ and nucleoside_1_ (N_1_)-2′-*O* positions (Grdzelishvili et al., 2005; Li et al., 2005, 2006; Ogino et al., 2005; Paesen et al., 2015; Martin et al., 2018). As suggested for vaccinia virus N_1_-2′-*O*-MTase (VP39), the second lysine residue in the K–D–K–E tetrad may play a critical role in cap-specific N_1_-2′-*O*-methylation by steering the 2′-OH oxygen orbital toward the methyl group of SAM (Li et al., 2004) rather than by directly deprotonating the 2′-OH group (Hager et al., 2002).

## Mechanisms of Cellular and Viral mRNA Capping

The 5′-terminal cap structure was discovered in viral mRNAs (Furuichi and Miura, 1975; Furuichi et al., 1975a; Wei and Moss, 1975) and subsequently in cellular mRNAs (Adams and Cory, 1975; Desrosiers et al., 1975; Furuichi et al., 1975b; Perry and Kelley, 1975) as a universal blocked structure of eukaryotic mRNAs in 1975. The cap structure is composed of *N*^7^-methylguanosine (m^7^G) linked to the first nucleoside (N_1_) of mRNA through an inverted 5′-5′ triphosphate bridge (ppp). Lower eukaryotic mRNAs have an m^7^GpppN_1_- cap structure (called cap 0), whereas the cap structure of higher eukaryotic mRNAs is further methylated at 2′-*O* positions to various degrees into m^7^GpppN_1_m- (cap 1) and m^7^GpppN_1_mpN_2_m- (cap 2) [reviewed in Banerjee (1980)]. In all eukaryotic cells, the positively charged m^7^G moiety is required for mRNA biogenesis and metabolism at various steps, such as mRNA stability, splicing, transport, and translation (reviewed in Banerjee, 1980; Furuichi and Shatkin, 2000; Ramanathan et al., 2016). To utilize such cellular cap-dependent systems, many viruses have established their own mRNA capping system. In higher eukaryotes, ribose-2′-*O*-methylation of the N_1_ residue in the cap structure of cellular mRNAs not only makes mRNAs more stable (Picard-Jean et al., 2018), but also provides them with a molecular signature to manifest as self mRNA (cap 1-RNA) differently from non-self mRNA (cap 0-RNA). Cap 0-RNA triggers innate immune reactions via non-self RNA sensors, such as RIG-I (retinoic acid-inducible gene I) (Schuberth-Wagner et al., 2015; Devarkar et al., 2016) and MDA5 (melanoma differentiation-associated gene 5) (Zust et al., 2011). Furthermore, translation of cap 0-RNA is inhibited by IFIT1 (interferon-induced with tetratricopeptide repeats 1), a cap 0 RNA-binding protein, and/or its related proteins (Daffis et al., 2010; Habjan et al., 2013; Kumar et al., 2014; Daugherty et al., 2016; Abbas et al., 2017; Johnson et al., 2018). To evade these innate immune reactions, many higher eukaryotic viruses have acquired their own cap-specific N_1_-2′-*O*-methylation system as well.

**FIGURE 7:**
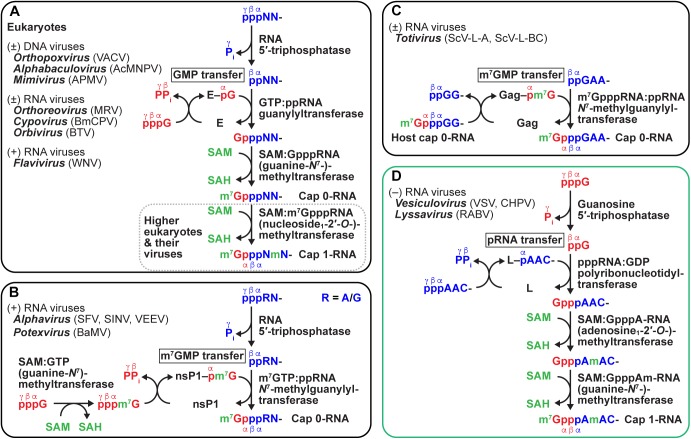
Diverse mechanisms of eukaryotic and viral mRNA cap formation. Conventional **(A)** and unconventional **(B–D)** pathways of eukaryotic and viral mRNA cap formation are schematically represented (for detail, see text). Transferases are expressed by their systematic names, in which their donor and acceptor substrates, separated by a colon, are included (for general names, see text and Table 1). GTP, pre-mRNA (5′-end only), and *S*-adenosyl-L-methionine (SAM) substrates are shown in red, blue, and, green, respectively. P_i_ and PP_i_ indicate inorganic phosphate and pyrophosphate, respectively. SAH denotes *S*-adenosyl-L-homocysteine. In **(A)**, guanylyltransferase is indicated by E (enzyme). In **(B–D)**, viral nucleotidyltransferases are expressed as respective protein names (nsP1, Gag, and L). Virus names (abbreviations) are as follows: vaccinia virus (VACV), *Autographa californica* multiple nucleopolyhedrovirus (AcMNPV), *Acanthamoeba polyphaga* mimivirus (APMV), mammalian reovirus (MRV), *Bombyx mori* cypovirus (BmCPV), Bluetongue virus (BTV), West Nile virus (WNV), Semliki Forest virus (SFV), Sindbis virus (SINV), Venezuelan equine encephalitis virus (VEEV), bamboo mosaic virus (BaMV), *Saccharomyces cerevisiae* viruses L-A and L-BC (ScV-L-A, ScV-L-BC), Chandipura virus (CHPV).

In the nucleus of eukaryotic cells, the 5′-terminal cap structure [m^7^GpppN_1_(m)pN_2_(m)-] is sequentially formed on pre-mRNA by mRNA capping enzyme with the RNA 5′-triphosphatase (RTPase) and mRNA guanylyltransferase (GTase) activities followed by a cap-specific MTase(s) (reviewed in Furuichi and Shatkin, 2000; Shuman, 2001; Ghosh and Lima, 2010) (Figure 7A and Table 1). (1) RTPase hydrolyzes 5′-triphosphorylated RNA (pppRNA) into 5′-diphosphorylated RNA (ppRNA) and inorganic phosphate (P_i_). (2) GTase transfers a GMP moiety of GTP (GMP donor) to ppRNA (GMP acceptor) via a covalent enzyme-(lysyl-*N*^ζ^)–GMP intermediate to form GpppN_1_-RNA with concomitant release of PP_i_. (3) mRNA (guanine-*N*^7^-)-methyltransferase (G-*N*^7^-MTase) transfers a methyl group from SAM to GpppN_1_-RNA to generate m^7^GpppN_1_-RNA (cap 0-RNA) and a byproduct, *S*-adenosyl-L-homocysteine (SAH). (4) In higher eukaryotic cells, mRNA (nucleoside_1_-2′-*O*-)-methyltransferase (N_1_-2′-*O*-MTase) methylates cap 0-RNA to yield m^7^GpppN_1_m-RNA (cap 1-RNA) (Belanger et al., 2010). (5) Furthermore, mRNA (nucleoside_2_-2′-*O*-)-methyltransferase (N_2_-2′-*O*-MTase) methylates cap 1-RNA to produce m^7^GpppN_1_mpN_2_m-RNA (cap 2-RNA) (Werner et al., 2011). (6) If the N_1_ residue of mRNA is 2′-*O*-methyladenosine, mRNA (2′-*O*-methyladenosine_1_-*N*^6^-)-methyltransferase (Am_1_-*N*^6^-MTase) often methylates the cap 1 structure to generate m^7^Gpppm^6^Am-RNA (m^6^Am: *N*^6^,2′-*O*-dimethyladenosine) (Wei et al., 1975; Akichika et al., 2019). m^6^A methylation of the cap 1 structure may regulate stability and/or translation of a subset of mRNAs (Mauer et al., 2017; Akichika et al., 2019), although there is currently no consensus on its role(s) (Shi et al., 2019).

**Table 1:** Enzymes involved in mRNA cap formation.

EC number	Enzyme name [systematic name^a^]	Abbreviation	Protein (species/virus^b^)
3.6.1.^c^	RNA 5′-triphosphatase	RTPase	RNGTT/HCE (human), D1 (VACV), λ1/μ2 (MRV), NS3 (WNV), nsP2 (SFV)
2.7.7.50	mRNA guanylyltransferase [GTP:ppRNA guanylyltransferase]	GTase	RNGTT/HCE (human), D1 (VACV), λ2 (MRV), NS5 (WNV)
2.1.1.56	mRNA (guanine-*N*^7^-)-methyltransferase [SAM:GpppN-RNA (guanine-*N*^7^-)-methyltransferase]	G-*N*^7^-MTase	RNMT/hMet (human), D1 (VACV), λ2 (MRV), NS5 (WNV), L (SeV)
2.1.1.56	mRNA (guanine-*N*^7^-)-methyltransferase [SAM:GpppNm-RNA (guanine-*N*^7^-)-methyltransferase]	G-*N*^7^-MTase	L (VSV, HMPV, SUDV)
2.1.1.57	mRNA (nucleoside_1_-2′-*O*-)-methyltransferase^d^ [SAM:m^7^GpppN-RNA (nucleoside_1_-2′-*O*-)-methyltransferase]	N_1_-2′-*O*-MTase	CMTR1/MTr1/ISG95 (human), VP39 (VACV), λ2 (MRV), NS5 (WNV)
2.1.1.57	mRNA (nucleoside_1_-2′-*O*-)-methyltransferase [SAM:GpppN-RNA (nucleoside_1_-2′-*O*-)-methyltransferase]	N_1_-2′-*O*-MTase	L (VSV, HMPV, SUDV)
2.1.1.296	mRNA (nucleoside_2_-2′-*O*-)-methyltransferase^e^ [SAM:m^7^GpppNm-RNA (nucleoside_2_-2′-*O*-)-methyltransferase]	N_2_-2′-*O*-MTase	CMTR2/MTr2 (human)
2.1.1.62	mRNA (2′-*O*-methyladenosine_1_-*N*^6^-)-methyltransferase [SAM:m^7^GpppAm-RNA (2′-*O*-methyladenosine_1_-*N*^6^-)-methyltransferase]	Am_1_-*N*^6^-MTase	CAPAM/PCIF1 (human)
2.1.1.^c^	GTP (guanine-*N*^7^-)-methyltransferase [SAM:GTP (guanine-*N*^7^-)-methyltransferase]	G-*N*^7^-MTase	nsP1 (SFV, SINV, VEEV), Replicase (BaMV)
2.7.7.^c^	mRNA *N*^7^-methylguanylyltransferase [m^7^GTP:ppRNA *N*^7^-methylguanylyltransferase]	m^7^GTase	nsP1 (SFV, SINV, VEEV), Replicase (BaMV)
2.7.7.^c^	mRNA *N*^7^-methylguanylyltransferase [m^7^GpppN-RNA:ppRNA *N*^7^-methylguanylyltransferase]	m^7^GTase	Gag (ScV-L-A)
3.6.1.^f^	guanosine 5′-triphosphatase/nucleoside 5′-triphosphatase	GTPase/NTPase	L (VSV, RABV, HMPV)
2.7.7.88	GDP polyribonucleotidyltransferase [pppRNA:GDP polyribonucleotidyltransferase^g^]	PRNTase	L (VSV, CHPV, RABV)


*^a^In the first part of systematic names for transferases, donor and acceptor substrates, separated by a colon, are shown. pp, 5′-diphospho; GpppN, guanosine(5′)triphospho(5′)nucleoside (cap core); GpppNm, guanosine(5′)triphospho(5′)2′-*O*-methylnucleoside; m^*7*^GpppN, *N*^*7*^-methylguanosine(5′)triphospho (5′)nucleoside (cap 0); m^*7*^GpppNm, *N*^*7*^-methylguanosine(5′)triphospho(5′)2′-*O*-methylnucleoside (cap 1); SAM, *S*-adenosyl-L-methionine; m^*7*^GTP, *N*^*7*^-methylguanosine-5′-triphosphate; ppp, 5′-triphospho. ^*b*^VACV, vaccinia virus; MRV, mammalian reovirus; WNV, West Nile virus; SFV, Semliki Forest virus; SeV, Sendai virus; VSV, vesicular stomatitis virus; HMPV, human metapneumovirus; SUDV, Sudan virus; BaMV, bamboo mosaic virus; ScV-L-A, *Saccharomyces cerevisiae* virus L-A; RABV, rabies virus; CHPV, Chandipura virus. ^*c*^Not assigned. ^*d*^Accepted name: methyltransferase cap1. ^*e*^Accepted name: methyltransferase cap2. ^*f*^Suggested number: 3.6.1.15 (nucleoside-triphosphate phosphatase). ^*g*^Accepted systematic name: 5′-triphospho-mRNA:GDP 5′-phosphopolyribonucleotidyltransferase.*

Nucleocytoplasmic large DNA viruses [e.g., vaccinia virus (Ensinger et al., 1975; Wei and Moss, 1975; Venkatesan et al., 1980; Shuman and Hurwitz, 1981), baculovirus (Gross and Shuman, 1998), mimivirus (Benarroch et al., 2008)], higher eukaryotic dsRNA viruses [e.g., reovirus (Furuichi et al., 1976; Cleveland et al., 1986; Bisaillon and Lemay, 1997; Kim et al., 2004), cytoplasmic polyhedrosis virus (Furuichi and Miura, 1975; Shimotohno and Miura, 1976), Bluetongue virus (Martinez-Costas et al., 1998; Ramadevi et al., 1998)], and positive-strand RNA flaviviruses [e.g., West Nile virus (Ray et al., 2006; Issur et al., 2009)] follow the same pathway of eukaryotic mRNA capping (Figure 7A). In contrast, some positive-strand RNA viruses, such as alphaviruses [e.g., Semliki Forest virus (Ahola and Kääriäinen, 1995; Vasiljeva et al., 2000), Venezuelan equine encephalitis virus (Li et al., 2015)], and potexviruses [e.g., bamboo mosaic virus (Huang et al., 2005)], use a slightly different capping pathway (Figure 7B). (1) Alphaviral RTPase generates ppRNA from pppRNA. (2) GTP G-*N*^7^-MTase methylates GTP to produce m^7^GTP. (3) mRNA *N*^7^-methylguanylyltransferase (m^7^GTase) transfers m^7^GMP from m^7^GTP to ppRNA via a covalent enzyme–m^7^GMP intermediate to generate m^7^GpppN_1_-RNA (cap 0-RNA). Interestingly, although alphaviruses do not have N_1_-2′-*O*-MTase, a structural element within the 5′-untranslated region of their cap 0-RNA genome confers resistance to the IFIT1-mediated restriction (Hyde et al., 2014). On the other hand, yeast dsRNA totiviruses (e.g., *Saccharomyces cerevisiae* viruses L-A and L-BC) have a new class of mRNA m^7^GTases. Totiviral m^7^GTase transfers an m^7^GMP moiety of host m^7^GpppRNA to viral ppRNA through a covalent enzyme-(histidyl-*N*)-m^7^GMP intermediate to generate viral m^7^GpppN_1_-RNA, thereby causing decapping of host mRNAs (Figure 7C; Blanc et al., 1994; Fujimura and Esteban, 2011, 2012, 2013). The totiviral capping mechanism is called “cap-snatching,” but is significantly different from the cap-snatching mechanism involving cap-dependent endonuclease that was originally discovered in influenza virus (Plotch et al., 1981).

## Mechanisms of NNS RNA Viral mRNA Capping

The pioneering studies using the *in vitro* VSV transcription system demonstrated that the VSV-associated capping enzyme co-transcriptionally incorporates a GDP moiety of GTP into the cap core structure (Gpp-pA) of mRNAs (Abraham et al., 1975a,b), and two MTases methylate the cap structure at the adenosine_1_ (A_1_)-2′-*O* position followed by the G-*N*^7^ position to sequentially generate GpppAm and m^7^GpppAm (cap 1) (Testa and Banerjee, 1977). Therefore, the pathway of the cap 1 formation by the VSV system appeared to be significantly different from those by the host and other viral systems. However, the precise mechanisms of the cap formation had remained elusive for three decades due to the lack of an *in vitro* cap formation assay.

The development of a VSV capping system with an exogenously added oligo-RNA substrate (Ogino and Banerjee, 2007, 2008; Ogino et al., 2010; Ogino, 2013) led to the breakthrough in understanding the molecular mechanisms of rhabdoviral mRNA capping as well as identifying the L protein as a non-canonical mRNA capping enzyme (reviewed in Ogino and Banerjee, 2011a,b). Importantly, a recombinant form of the VSV L protein as well as native VSV L–P and RNP complexes was shown to catalyze the unique RNA capping reaction (Ogino and Banerjee, 2007). In striking contrast to the mononucleotide (GMP or m^7^GMP) transfer mechanisms employed by eukaryotes and other viruses (Figure 7A–C), rhabdoviruses, such as VSV (Ogino and Banerjee, 2007, 2008; Ogino et al., 2010), Chandipura virus (CHPV, *Vesiculovirus*) (Ogino and Banerjee, 2010), and RABV (*Lyssavirus*) (Ogino et al., 2016; Ogino and Green, 2019), use a polynucleotide transfer mechanism to generate the cap core structure (Figure 7D). In the first step of the cap formation, the L protein-associated guanosine 5′-triphosphatase (GTPase) activity removes the γ-phosphate of GTP to generate GDP (Ogino and Banerjee, 2007, 2008). GDP polyribonucleotidyltransferase (PRNTase) (the L protein) transfers a pRNA moiety of pppRNA (pRNA donor) to GDP (pRNA acceptor) via a covalent enzyme (L)-(histidyl-*N*^ε2^)–pRNA (L–pRNA) intermediate to yield GpppA-RNA in an mRNA-start sequence dependent manner (Ogino and Banerjee, 2007; Ogino et al., 2010). PRNTase can also transfer pRNA to GTP to produce a small amount of a tetraphosphate-containing cap structure, G(5′)pppp(5′)A, as a byproduct although to a lesser extent than GpppA (Ogino and Banerjee, 2008). However, it is likely that VSV can bypass the GTP hydrolysis step for the formation of GpppA on VSV mRNAs in infected cells, because infected cells may contain sufficient concentrations of GDP (Traut, 1994) that are three to four orders of magnitude higher than the *K*_m_ for GDP (0.03 μM) (Ogino and Ogino, 2017). In addition, the L–pRNA intermediate is able to transfer pRNA to PP_i_ to produce pppRNA (Ogino et al., 2010), although less efficiently than to GDP to form GpppRNA, indicating that the step of the intermediate formation with pppRNA is reversible.

Consistent with the 5′-end states of VSV mRNAs and LeRNA (Abraham et al., 1975a,b; Colonno and Banerjee, 1976), the VSV L protein (PRNTase) caps pppRNAs with the vesiculoviral mRNA start-sequence (5′-AACAG), but not the LeRNA start-sequence (5′-ACGAA), by specifically recognizing the former sequence at the step of the covalent L–pRNA intermediate formation (Ogino and Banerjee, 2007, 2008; Ogino, 2013). Mutagenesis studies identified the 5′-ARCNG (R = A or G) sequence as the VSV mRNA capping signal, in which the first three residues (A_1_R_2_C_3_) and the fifth residue (G_5_) are essential and important, respectively, for the pRNA donor substrate activity (Ogino and Banerjee, 2007, 2008). The efficiency of the capping reaction increases with the increase of the chain length of the mRNA-start sequence from four to 6 nt in the intermediate formation step (Ogino, 2013). In sharp contrast to eukaryotic capping enzyme (Venkatesan and Moss, 1980), the VSV L protein is able to cap pppRNAs, but not ppRNAs, with GDP (Ogino and Banerjee, 2007). Similarly, the RABV L protein specifically caps pppRNAs, but not ppRNAs, with the lyssaviral mRNA-start sequence 5′-AACA(C/U), in which the 5′-terminal AAC sequence is critical for the substrate activity (Ogino et al., 2016; Ogino and Green, 2019). Unlike the VSV L protein, the RABV L protein does not accept pppAGC-RNA as a pRNA donor substrate (Ogino et al., 2016).

Using the VSV *in vitro* transcription system, it was shown that virion-associated A_1_-2′-*O*- and G-*N*^7^-MTases with lower (0.5 μM) and higher (10 μM) *K*_m_ values for SAM, respectively, co-transcriptionally methylate the cap structure on mRNAs, producing GpppAm in the presence of limited concentrations (< 0.1 μM) of SAM and m^7^GpppAm (along with GpppAm) in the presence of higher concentrations (> 5 μM) of SAM (Testa and Banerjee, 1977). In addition, pulse-chase experiments demonstrated that GpppAm-capped pre-mRNAs serve as precursors for m^7^GpppAm-capped pre-mRNAs, indicating the unique order of cap methylation: GpppA → GpppAm → m^7^GpppAm (Testa and Banerjee, 1977). It is interesting to note that vesicular stomatitis New Jersey virus carries out co-transcriptional cap methylation via major (GpppA- → GpppAm- → m^7^GpppAm-) and minor (GpppA- → m^7^GpppA- → m^7^GpppAm-) pathways (Hammond and Lesnaw, 1987). Since the VSV-associated MTases could not use exogenously added unmethylated VSV mRNAs as methyl acceptors, the MTase reactions had been thought to be tightly coupled to mRNA synthesis (Banerjee, 1980). Rahmeh et al. (2009) reported that a large amount (2 μg, ∼8 pmol) of a recombinant form of the VSV L protein alone can methylate GpppA on an oligo-RNA having the 10-nt VSV mRNA-start sequence at the A_1_-2′-*O* position followed by the G-*N*^7^ position to produce ∼10 fmol of m^7^GpppAm within 2 h. Nevertheless, our recombinant VSV L protein as well as a native L–P complex showed no MTase activity when using an exogenously added capped RNA substrate with the VSV *N* mRNA-start sequence (GpppAACAGUAAUC) under the reported conditions, although it was fully capable to generate m^7^GpppAm on mRNAs co-transcriptionally when using our reconstituted transcription system (Ogino, 2013). The reason for this discrepancy is currently not known.

Interestingly, the addition of SAH, the byproduct of the MTase reactions, to VSV *in vitro* transcription reactions is known to induce production of mRNAs with an extremely long poly(A) tail (Rose et al., 1977). Similarly, some mutations in the MTase domain (D1762E, K1651A) (Galloway and Wertz, 2008; Li et al., 2009) as well as the CD (F1488S) (Hunt and Hutchinson, 1993) of the VSV L protein induce hyperpolyadenylation of mRNAs independently of SAH. In contrast, other methylation-defective mutations (e.g., D1762G, G1672P, G1675P) in the VSV L protein render it unresponsive to SAH, producing normally polyadenylated mRNAs with or without SAH (Galloway and Wertz, 2008). These observations suggest that the MTase domain regulates mRNA 3′-polyadenylation by a mechanism not yet understood.

On the other hand, it was demonstrated that a small amount (3 ng, ∼12 fmol) of a recombinant form of the SeV (*Paramyxoviridae*) L protein is enough to specifically methylate a capped 5-nt RNA with the SeV mRNA-start sequence (GpppAGGGU) at the G-*N*^7^ position, but not at the A_1_-2′-*O* position, to produce ∼10 fmol of m^7^GpppA within 2 h (Ogino et al., 2005). A C-terminal part of the recombinant SeV L protein alone exhibits the G-*N*^7^-MTase activity although to a ∼100-fold lesser extent than the full-length SeV L protein (Ogino et al., 2005). Since native SeV RNPs co-transcriptionally generate m^7^GpppAm along with m^7^GpppA on *in vitro* synthesized mRNAs (Takagi et al., 1995), the order of cap methylation by SeV appears to be different from that by VSV and to be as follows: GpppA → m^7^GpppA → m^7^GpppAm. Similarly, HRSV RNPs produce m^7^GpppG and m^7^GpppGm on mRNAs during *in vitro* transcription in the presence of lower and higher concentrations of SAM, respectively (Barik, 1993; Liuzzi et al., 2005), suggesting that G-*N*^7^-methylation precedes guanosine_1_ (G_1_)-2′-*O*-methylation similar to eukaryotic cap methylation. In contrast, recombinant C-terminal fragments of the L proteins of human metapneumovirus (HMPV) (Paesen et al., 2015), closely related to HRSV, and Sudan virus (SUDV, *Filoviridae*) (Martin et al., 2018) were reported to methylate the cap structure at the G_1_-2′-*O* position followed by the G-*N*^7^ position, although cap methylation activities of either recombinant or native forms of their full-length L proteins have not been characterized. Therefore, there seem to be variations in the order of cap methylation among NNS RNA viruses. Intriguingly, the C-terminal fragment of the SUDV L protein methylates internal adenosine residues at the 2′-*O* position in oligo-RNAs *in vitro* (Martin et al., 2018), although there is currently no evidence that internal adenosine residues in viral and/or host RNAs are methylated with the SUDV L protein in infected cells.

The cap structure of VSV mRNAs isolated from infected cells is known to be more extensively methylated into m^7^Gppp(m^6^)A_1_mp(m^6^)A_2_(m)-, where A_1_ is predominantly m^6^Am and A_2_ is A, Am, or m^6^Am (Moyer et al., 1975; Moyer and Banerjee, 1976). In contrast, *N*^6^-methylation of A_1_ and A_2_ and 2′-*O*-methylation of A_2_ of the cap structure do not occur on mRNAs synthesized with detergent-disrupted VSV *in vitro* (Abraham et al., 1975b). Thus, cellular cap-specific Am_1_-*N*^6^- and N_2_-2′-*O*-MTases appear to be involved in these additional methylation reactions to VSV mRNAs in infected cells. No information is currently available on types of cap structure on other NNS RNA viral mRNAs produced in infected cells.

## GDP Polyribonucleotidyltransferase

**FIGURE 8:**
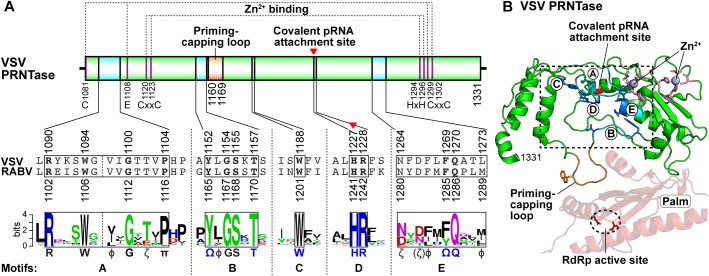
Structure of the PRNTase domain in the VSV L protein. **(A)** The VSV PRNTase domain is schematically represented with the conserved motifs A–E (cyan) and priming-capping loop (orange). The covalent pRNA attachment site (Ogino et al., 2010) is indicated by a red arrowhead. The positions of amino acid residues responsible for binding to two Zn^2+^ ions are indicated. Sequence logos for PRNTase motifs A–E in L proteins of 227 NNS RNA viruses belonging to the *Rhabdoviridae, Paramyxoviridae, Filoviridae, Bornaviridae*, and *Nyamiviridae* families (excluding novirhabdoviruses) (Maes et al., 2019) are shown with the corresponding sequences of VSV and RABV. Φ and π denote hydrophobic and small amino acids, respectively (other symbols, see Figure 5). **(B)** The three-dimensional structure of the PRNTase domain in the VSV L protein (PDB id: 5A22) is shown as a ribbon diagram (green). The PRNTase motifs A–E (labeled by circled letters), priming-capping loop, and Zn^2+^-binding sites are colored cyan, orange, and pink, respectively. Key amino acid residues (T1152, T1157, W1188, H1227, R1228, F1269, and Q1270) are depicted as stick models (blue carbon backbone). Zinc ions are shown as light blue spheres. A close-up view of the PRNTase active site within the dashed box is shown in Figure 9A. The RdRp palm subdomain is shown in pale red with the catalytic aspartate residues (D605 and D714, red stick models within the dashed ellipse).

The VSV PRNTase domain is composed of 251 amino acid residues and contains five highly conserved amino acid sequence elements, Rx(3)Wx(3–8)ΦxGxζx(P/A) (motif A; Φ, hydrophobic), (Y/W)ΦGSxT (motif B), W (motif C), HR (motif D), and ζxxΦx(F/Y)QxxΦ (motif E) (Figure 8A; Neubauer et al., 2016). These motifs were identified by aligning amino acid sequences of more than 200 L proteins of NNS RNA viruses belonging to the *Rhabdoviridae, Paramyxoviridae, Pneumoviridae, Filoviridae, Bornaviridae*, and *Nyamiviridae* families (Ogino and Banerjee, 2011a,b; Neubauer et al., 2016). Motifs B–E are located in close proximity in the center of the flat domain (Liang et al., 2015; Neubauer et al., 2016; Figure 8B), forming an active site of the enzyme. A helix-loop structure that contains motif A may play structural roles in providing a platform for the PRNTase active site organization and/or connecting the PRNTase domain to the bridge domain. Two cysteine/histidine-rich Zn^2+^ coordinating elements (C1181–E1108–C1299–C1302 and C1120–C1223–H1292–H1296) are present in the VSV PRNTase domain (Liang et al., 2015), but are not fully conserved in other viral PRNTase-like domains (Ogino and Banerjee, 2011a). The domain also possesses a characteristic large loop structure flanking PRNTase motif B (called “priming-capping loop”) (Ogino and Green, 2019; Ogino et al., 2019), which is deeply inserted into the active site cavity of the RdRp domain (Liang et al., 2015).

Four conserved amino acid residues (G1154 and T1157 in motif B; H1227 and R1228 in motif D, also called GxxT[n]HR motif) of the VSV L protein were originally identified as essential for the formation of a product sensitive to tobacco acid pyrophosphatase, most probably 5′-terminal GpppA cap, on RNA by alanine scanning mutagenesis (Li et al., 2008), although this study did not address which step(s) of capping is impaired by alanine mutations of these residues. To locate the active site of the VSV PRNTase domain, we precisely mapped a covalent pRNA attachment site in the VSV L protein (Ogino et al., 2010). After the formation of the L–pRNA (pAACAG) intermediate, it was enzymatically digested into a peptide-AMP complex. The peptide-AMP complex with an acid-labile bond was successfully isolated under neutral pH conditions, and analyzed by MALDI-TOF tandem mass spectrometry. These mass spectrometric and other biochemical analyses conclusively revealed that the 5′-terminal phosphate of the RNA is linked to the *N*^ε2^ position of H1227 in the VSV PRNTase domain via a phosphoamide bond (Ogino et al., 2010). H1227 is part of the histidine-arginine (HR) motif (motif D), which is critical for the pRNA transfer reaction in the step of the covalent intermediate formation, but not for GTP hydrolysis into GDP, during the cap formation (Ogino et al., 2010). A proposed role of the GxxT motif in guanosine nucleotide binding (Liang et al., 2015) requires experimental evidence.

**FIGURE 9:**
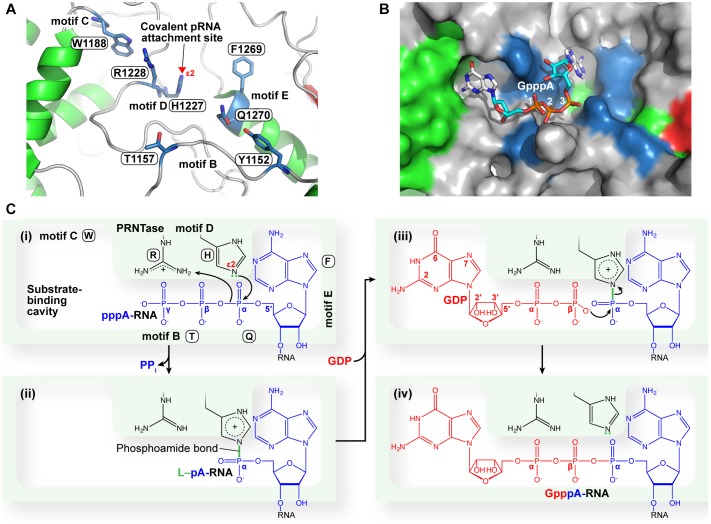
A proposed catalytic mechanism of mRNA capping by the PRNTase domain of the VSV L protein. **(A)** The three-dimensional structure of the VSV PRNTase active site (PDB id: 5A22) is shown as a ribbon diagram, in which α-helices, β-sheets, and loops are colored green, red, and gray, respectively. The key amino acid residues in PRNTase motifs B–E (Neubauer et al., 2016) are shown as stick models (blue carbon backbone). The covalent pRNA attachment site (*N*^ε2^ atom) of catalytic H1227 in motif D (Ogino et al., 2010) is indicated by a red arrowhead. The side-chain carbonyl group of Q1270 in motif E interacts with the hydroxyl group of Y1152 in motif B via hydrogen-bonding. **(B)** A surface representation of the view in **(A)** is shown with a docked GpppA 5′-cap analog in stick model. The GpppA resides in a crevice heavily generated by motifs B–E. Docking was performed with Autodock Vina (Trott and Olson, 2010). **(C)** The 5′-pppA residue of VSV pre-mRNA may dock into one side of a putative substrate-binding cavity surrounded by motifs E, D, and B **(i)**, where a nucleophile formed on the *N*^ε2^ position of H1227 subsequently attacks the α-phosphorus in the 5′-triphosphate group of pre-mRNA to form the covalent L–pRNA intermediate **(ii)**. GDP may dock into another side of the putative substrate-binding pocket surrounded by motifs C, D, and B **(iii)**. There, an oxyanion on the β-phosphate of GDP nucleophilically attacks the α-phosphorus of pRNA linked to H1227, resulting in the formation of the GpppA cap structure on pre-mRNA **(iv)**.

Our extensive mutagenesis analysis further identified G1100 in motif A, T1157 in motif B, W1188 in motif C, and F1269 and Q1270 in motif E as essential or important for the PRNTase activity in the step of the intermediate formation (Neubauer et al., 2016). These key residues as well as the catalytic residues in motif D are crucial for VSV gene expression and growth in cultured cells (Ogino, 2014; Neubauer et al., 2016). In the three-dimensional structure of the VSV L protein, these key residues surround the catalytic HR motif to form the unique active site of the PRNTase domain (Figure 9A; Liang et al., 2015; Neubauer et al., 2016). Since the G1154A mutation in motif B renders the VSV L protein more insoluble and inert in all the examined enzymatic reactions (capping, RNA synthesis, and GTP hydrolysis) (Neubauer et al., 2016), G1154 is likely to be critical for proper folding of the entire protein. Furthermore, some mutations (e.g., P1104A, Y1152A, Y1152W, L1153A, W1188A, and Q1270N) significantly reduce its RNA synthesis activity in addition to the PRNTase activity (Neubauer et al., 2016), suggesting that these mutations may also affect the folding of its local or entire structure to some extents. The hydroxyl group of Y1152 is hydrogen-bonded to the side-chain carbonyl group of Q1270 (Neubauer et al., 2016), suggesting that the interaction between these residues plays a structural role in forming the PRNTase active site.

To speculate functions of the VSV PRNTase domain in the cap formation, we performed a docking study with a GpppA cap analog corresponding to a 5′-terminal part of a capped RNA product (GpppAACAG-) and found that it fits in a crevice adjacent to motifs B–E (Figure 9B). The guanine base of the cap structure sits in a side cavity that is lined on one side with W1188. This base is positioned to interact with the side chain of R1221 and main chain atoms from R1181, D1184, and S1230. The 2′-OH of the ribose sits adjacent to the main chain carbonyl of R1228, while the 3′-OH appears to interact with the main chain carbonyl of R1233. The terminal oxygen of the side chain of S1230 sits within 3 angstrom of both the 2′- and 3′OH. The first phosphate lies between R1228 and K1156. Phosphate two is positioned to have an interaction with the *O*^γ1^ atom of T1157, while phosphate three is positioned to interact with the side chain of Q1270. The adenine base punches into a second cavity on the opposite side of the cleft from the guanine. F1269 sits to one side of the entrance to this cavity and is in position where by rotation of the side chain could π-stack with the adenine. The adenine base is also positioned ∼3 angstroms from the side chains of N1264 and S1224, which could interact with atoms *N*^6^ and *N*^7^, respectively. The active site histidine, H1227, sits between phosphate two and three of the cap analog.

Based on these observations, we suggest that the cavity serves as binding sites for the PRNTase substrates and products in the two-step ping-pong reaction. In the first step, the 5′-pppA residue of VSV pre-mRNA may reside within the right side of the cavity, which is constituted by amino acid residues, in part, in motifs B (e.g., S1155, T1157), D (H1227 and R1228), and E (e.g., N1264, D1266, L1268, F1269, Q1270) (Figure 9Ci). The aromatic side chain of F1269 may bind the adenine ring of the 5′-pppA residue via a stacking interaction. Other aromatic amino acids (Y or W) can substitute for F1269 *in vitro* RNA capping as well as replication of recombinant VSV in host cells (Neubauer et al., 2016). However, it is currently not known how the 5′-AAC sequence in pre-mRNA is recognized with the PRNTase domain in a sequence-dependent manner. T1157 and R1228 appear to be involved in recognition of the terminal γ and/or β-phosphate(s) of the RNA to form a non-covalent complex with pppRNA. Subsequently, a lone pair of electrons at the *N*^ε2^ position of H1227 nucleophilically attacks the α-phosphorus in the 5′-pppA residue of the RNA, resulting in the L–pRNA intermediate formation (Ogino et al., 2010) (Figure 9Cii). Simultaneously, a proton may be transferred from an amino acid residue serving as a proton donor (general acid) to a leaving PP_i_ group. R1228 plays a critical role(s) in the intermediate formation step, and can be replaced with histidine yielding partial activity, but not with lysine, in the pRNA transfer reaction (Ogino et al., 2010). The basic nature of R1228 suggests that its positively charged guanidino group is required for binding to the 5′-triphosphate group of the RNA and/or possibly the putative proton transfer to leaving PP_i_.

In the second step of the pRNA transfer reaction, GDP may be positioned in the left side of the cavity (Figure 9Ciii), if it is not occupied in the L–pRNA intermediate. W1188 (motif C) and less- or non-conserved amino acid residues (e.g., R1221) in a loop structure containing motif D create this putative GDP binding site. However, mutations of W1188 (W1188A, W1188F) and R1221 (R1221A, R1221H) in the VSV L protein were found not only to abolish their RNA capping activity in the step of the intermediate formation, but also to diminish RNA synthesis activity (Ogino et al., 2010; Ogino, 2014; Neubauer et al., 2016), suggesting that these mutations may have impacts on the folding of the entire PRNTase domain as well as the whole L protein. Since no mutations in the putative GDP binding site that affect the pRNA transfer to GDP, rather than the L–pRNA intermediate formation, have been found, it is currently not clear whether the putative GDP binding site is specifically required for the transfer reaction. Furthermore, we suggest that R1228 and T1157 may interact with the α and/or β-phosphate(s) of GDP. Finally, the PRNTase domain transfers pRNA from the L–pRNA intermediate to GDP, but not to other NDPs, to generate the GpppA cap structure on the RNA (Ogino et al., 2010) (Figure 9Civ). Biochemical studies (Ogino et al., 2010; Ogino and Ogino, 2017) indicate that the *C*^2^-amino group of guanine and 2′ or 3′-hydroxyl group of ribose in GDP are essential for the pRNA transfer reaction, while the *C*^6^-oxo group, *N*^1^-hydrogen, and *N*^7^-nitrogen are dispensable. Furthermore, m^7^GDP and 8-iodo-GDP serve as efficient pRNA acceptors to form cap structures (Ogino and Ogino, 2017). This result suggests that the PRNTase domain has an ample space for the *N*^7^-methyl or *C*^8^-iodo group on GDP, and is consistent with the docking model of the PRNTase domain with GpppA (Figure 9B), in which the *N*^7^ and *C*^8^ positions of the guanine ring are exposed to solvent. Although R1221 was predicted to be associated with the *C*^6^-oxo group of the guanine ring in the docking model with GpppA, the *C*^6^-oxo group in GDP is not necessary for the transfer reaction (Ogino and Ogino, 2017). Therefore, it still remains elusive which amino acid residues in the PRNTase domain specifically recognize GDP.

## Roles of the PRNTase Domain in Transcription

**FIGURE 10:**
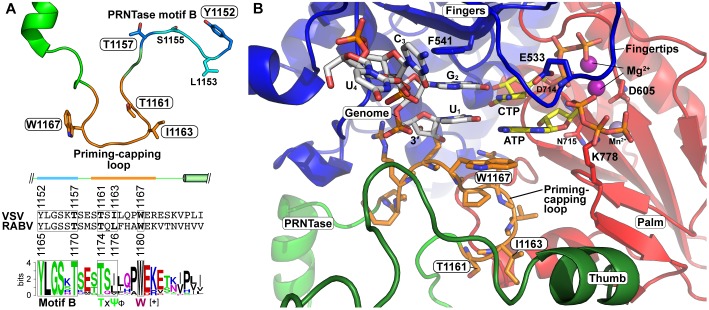
Formation of the VSV terminal *de novo* initiation complex. **(A)** The three-dimensional structural model of the priming-capping loop (residues 1160–1169, orange), with its flanking regions including PRNTase motif B, of the VSV L protein is represented as a ribbon diagram with stick models of key amino acid residues (Ogino et al., 2019) (upper). An amino acid sequence logo for putative priming-capping loops and their franking sequences of 110 vertebrate and arthropod rhabdoviruses (Ogino et al., 2019) are shown with the corresponding sequences of VSV and RABV (lower). The secondary structures of this region in the VSV L protein are depicted above its sequence. For amino acid symbols, see Figure 5A. **(B)** The structure of the VSV L protein in complex with the 3′-terminal sequence (3′-UGCU-5′) of the genome (white carbon backbone), initial (ATP) and incoming (CTP) nucleotides (yellow carbon backbone), two Mg^2+^ ions (purple), and Mn^2+^ ion (obscured) was modeled as described in Ogino et al. (2019) (Model Archive id: ma-5k432). W1167 on the priming-capping loop (orange carbon backbone) π-stacks with the initiator ATP. Key amino acid residues are shown as stick models on the fingers and palm subdomains. The RdRp subdomains and PRNTase/priming loop are colored as in Figure 5.

There is increasing evidence that the PRNTase domain regulates RNA synthesis in different steps. The VSV PRNTase domain has the dual-functional priming-capping loop (1160–1169) extended from the PRNTase domain into the RNA exit cavity of the RdRp domain (Liang et al., 2015; Ogino et al., 2019). The priming-capping loop of the L proteins of vertebrate and/or arthropod rhabdoviruses (e.g., VSV, CHPV, and RABV) contains a highly conserved tryptophan residue (VSV, W1167; RABV, W1180) and TxΨ motif (VSV, T1161-x-I1163; RABV, T1174-x-L1176) (Ogino and Green, 2019; Ogino et al., 2019; Figure 10A). The tryptophan residue is critical for terminal *de novo* transcription initiation, but not for internal *de novo* initiation, elongation, or capping (Ogino et al., 2019). In contrast, the TxΨ motif is required for capping in the step of the intermediate formation, but not for *de novo* transcription initiation, similar to the active site residues of the PRNTase domain (Ogino et al., 2019).

*De novo* initiating RdRps of other unrelated viruses (e.g., Φ6 phage, hepatitis C virus, reovirus, dengue virus, influenza virus) often have a “priming loop,” which facilitates primer-independent transcription initiation with initiator and incoming nucleotides by stabilizing their initiation complex formed at the 3′-terminal of their genomic RNAs (Butcher et al., 2001; Tao et al., 2002; Selisko et al., 2012; Appleby et al., 2015; Te Velthuis et al., 2016). Each priming loop is extended from a different position in a thumb or palm subdomain into their RdRp active sites and exhibits structural diversity. Different viruses use a distinct amino acid residue (e.g., tyrosine, serine, histidine, proline) as a priming amino acid to interact with a purine ring or triphosphate group of an initiator nucleotide (ATP or GTP) (Butcher et al., 2001; Tao et al., 2002; Selisko et al., 2012; Appleby et al., 2015; Te Velthuis et al., 2016).

Similar to other viral priming loops, the priming-capping loop extended from the PRNTase domain of rhabdoviral L proteins facilitates transcription initiation with initiator and incoming nucleotides (ATP and CTP, respectively) at the 3′-terminal UG sequence of the *Le* promoter in the genome (Ogino et al., 2019). Based on the structures of the terminal initiation complex of the phage Φ6 RdRp (PDB id: 1HI0) (Butcher et al., 2001) and the apo state of the VSV L protein (PDB id: 5A22) (Liang et al., 2015), the VSV terminal initiation complex containing ATP, CTP, and a 3′-UGCU template (3′-terminal sequence of the VSV genome) was modeled (Model Archive id: ma-5k432) (Ogino et al., 2019; Figure 10B). In this model, the bases of the initiator ATP and incoming CTP are aligned with the complementary 3′-UG sequence in the template RNA by Watson-Crick base pairing and the α-phosphate of CTP is associated with a Mg^2+^ ion adjacent to D714 in motif C. The adenine ring of ATP is sandwiched between the cytosine ring of CTP and the indole side chain of the tryptophan residue on the priming-capping loop via π-stacking interactions. Similar to the aromatic tyrosine residue on the priming loop of the Φ6 phage RdRp (Butcher et al., 2001), the tryptophan residue of the VSV L protein may provide a key structural platform for the terminal initiation complex formation.

**FIGURE 11:**
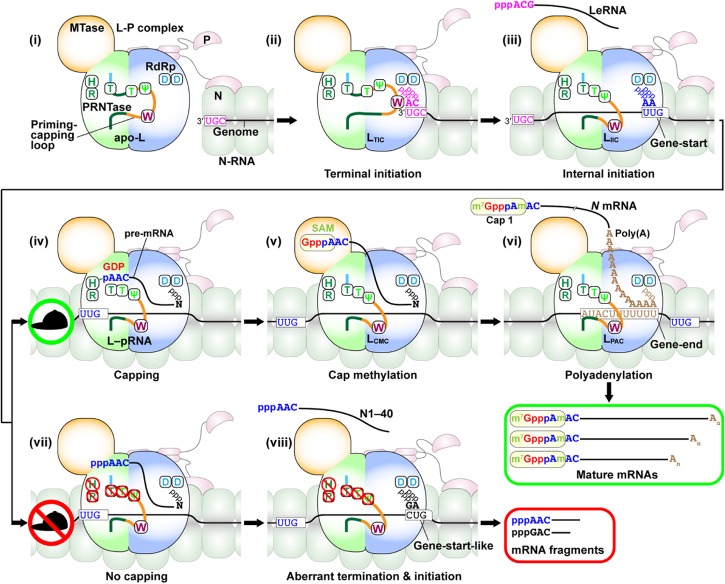
Transcriptional control by the PRNTase domain of the VSV L protein. The RdRp complex composed of the L and P proteins interacts with the N proteins located at the 3′-end of the genome using the C-terminal N–RNA binding domain of the P protein **(i)**. The RdRp domain of the L protein initiates *de novo* transcription with initiator ATP and incoming CTP on the 3′-terminal UG sequence of the genome **(ii)**. The tryptophan residue on the priming-capping loop of the PRNTase domain of the L protein is essential for terminal *de novo* initiation (see Figure 10). After synthesis of LeRNA, the RdRp complex reinitiates transcription with two ATP molecules at the internal *N* gene-start sequence without using the priming-capping loop **(iii)**. When the 5′-pppAAC end of *N* pre-mRNA reaches the active site of the PRNTase domain during mRNA chain elongation, the L protein forms the covalent L–pRNA intermediate **(iv)** and subsequently transfers pRNA from the intermediate to GDP to generate the GpppA cap core structure. The TxΨ motif on the priming-capping loop and key amino acid residues (e.g., T1157, H1227, and R1228) in PRNTase motifs (see Figure 8, 9) are required for capping in the step of the intermediate formation. After capping, the MTase domain of the L protein sequentially methylates the cap core structure at the adenosine-2′-*O* position followed by the guanine-*N*^7^ position into the cap 1 structure with concomitant mRNA chain elongation **(v)**. The RdRp domain of the L protein polyadenylates the 3′-end of full-length *N* mRNA by slippage at the U7 tract in the gene-end sequence **(vi)**. If the PRNTase domain fails to form the covalent L–pRNA intermediate during mRNA chain elongation **(vii)**, the RdRp domain frequently terminates transcription at an early stage of mRNA elongation, releasing 5′-triphosphorylated *N* pre-mRNA of 40 nt (N1–40), and carries out aberrant stop-start transcription using cryptic initiation and termination signals within the *N* gene, releasing a 28-nt RNA initiated with GTP **(viii)**. L_TIC_, L_IIC_, L_CMC_, and L_PAC_ indicate L complexes for terminal initiation, internal initiation, cap methylation, and polyadenylation, respectively.

In the first step of terminal transcription initiation (Figure 11i), binding of the C-terminal domain of the P protein to the N proteins on the 3′-terminal of the genome is likely to trigger their conformational change into an open state, which may lead to the access of the RdRp domain of the L protein in the RdRp complex to the 3′-UG sequence. Unlike a previous model for terminal *de novo* initiation (Leyrat et al., 2011a), the N^0^-binding region of the P protein is dispensable for this process (Ogino et al., 2019). As described above, the tryptophan residue on the priming-capping loop of the PRNTase domain may stabilize the terminal *de novo* initiation complex with ATP and CTP assembled on the 3′-UG sequence of the genome (Figure 11ii). Although this mechanism was proposed for *de novo* initiation to synthesize LeRNA (Ogino et al., 2019), the same mechanism should be used to initiate synthesis of the antigenome and genome from the 3′-termini of the genome and antigenome, respectively. The priming-capping loop is most likely to be retracted for RNA chain elongation, because it obstructs the RNA exit channel of the RdRp domain in the apo state of the VSV L protein (PDB id: 5A22) (Liang et al., 2015).

After synthesis of LeRNA, the same RdRp complex reinitiates transcription at the 3′-UUG sequence of the internal *N* gene-start sequence to generate *N* pre-mRNA (Figure 11iii). An *in vitro* transcription assay using oligo-RNA templates containing the internal *N* gene-start sequence for the RABV L protein revealed that the RABV counterpart (W1180) of the tryptophan residue (W1167) on the VSV priming-capping loop is not required for internal initiation (Ogino et al., 2019). The role of W1167 of the VSV L protein in internal initiation has not been directly investigated due to the unavailability of its internal initiation assay. Interestingly, a proline residue in a priming loop of the influenza virus RdRp is critical for terminal initiation at the 3′-end of the genomic promoter, but not for internal initiation within the anti-genomic promoter (Te Velthuis et al., 2016). These results suggest that the mechanism of internal *de novo* initiation by viral RdRps is different from that of terminal *de novo* initiation.

The 5′-pppAACAG end of VSV pre-mRNA extruded from the RNA exit channel of the RdRp domain gains access to the PRNTase domain during RNA chain elongation, and is specifically recognized with the domain to carry out the covalent L–pRNA intermediate formation followed by the pRNA transfer to GDP (Figure 11iv). In addition to the key amino acid residues in the PRNTase motifs (e.g., H1227, R1228, and T1157), the TxΨ motif adjacent the tryptophan residue in the priming-capping loop is critical for the formation of the L–pRNA intermediate (Ogino et al., 2019). Earlier studies have reported that VSV-associated RdRps generate uncapped (ppp- or pp-) (11–42 nt) and capped (23–41 nt) abortive transcripts with the 5′-AACAG sequence during *in vitro* transcription (Testa et al., 1980; Lazzarini et al., 1982; Pinney and Emerson, 1982; Schubert et al., 1982; Piwnica-Worms and Keene, 1983). Furthermore, using an *in vitro* reconstituted transcription system, it has been reported that the capping and two methylation reactions occur on a 31-nt, but not 30-nt, transcript when the VSV RdRp is artificially stalled at desired positions in genetically engineered genomes by omitting one of nucleotide substrates (Tekes et al., 2011). Although these studies suggest that the PRNTase domain of the VSV L protein caps pre-mRNAs at an early stage of mRNA chain elongation, none of them demonstrated whether these short RNAs are co-transcriptionally capped and methylated, or these capped short RNAs serve as precursors for full-length mRNAs. As described above, the cap structure of VSV pre-mRNA is sequentially and co-transcriptionally methylated at the two positions: GpppA- → GpppAm- → m^7^GpppAm- (Testa and Banerjee, 1977; Hammond and Lesnaw, 1987). During mRNA chain elongation, the single MTase domain of the L protein may carry out the two methylation reactions to generate the cap 1 structure (Figure 11v). Finally, the RdRp domain of the L protein adds a poly(A) tail to the 3′-end of full-length mRNA when transcribing the U tract in the gene-end sequence through a transcriptional slippage mechanism (Schubert et al., 1980; Iverson and Rose, 1981; Barr et al., 1997) (Figure 11vi).

The VSV RdRp can initiate mRNA synthesis from suboptimal initiation sequences (e.g., 3′-CUG, UGG) in the gene-start sequence, but prematurely terminates transcription, resulting in production of uncapped transcripts of 40–200 nt (Stillman and Whitt, 1999). Since 5′-GAC and ACC sequences of transcripts synthesized from the suboptimal initiation sequences do not match the 5′-ARC capping signal (Ogino and Banerjee, 2007, 2008), these transcripts are not able to form the L–pRNA intermediate during transcription. Some cap-defective mutations (e.g., H1227R, R1228H, T1157A, and Q1270A) in the VSV L protein, which abolish the L–pRNA intermediate formation, do not affect LeRNA synthesis, but frequently induce termination of *N* mRNA synthesis at the +40 (or +38) position, releasing a 5′-triphosphorylated abortive *N* mRNA fragment (called N1–40) (Ogino, 2014; Neubauer et al., 2016; Figure 11vii,viii). Therefore, the L–pRNA intermediate followed by the pRNA transfer to GDP seems to be a key step to determine whether the RdRp domain of the L protein continues to elongate mRNA chain or terminates transcription at the +40 position.

After releasing N1–40, these cap-defective mutants abnormally use cryptic initiation and termination signals within the *N* gene, producing unusual *N* mRNA fragments initiated with GTP (a non-canonical initiator nucleotide for the VSV RdRp), such as an internal fragment with residues 41–68 (N41–68) and 3′-polyadenylated fragment with residues 157–1326 (N157–1326) (Ogino, 2014; Neubauer et al., 2016). It should be noted that an early study using an *in vitro* transcription system with detergent-disrupted native VSV (Schubert et al., 1982) had already identified the cryptic transcription termination signal at position +40 to generate N1–40 and the cryptic initiation and termination signals at positions +41 and +68, respectively, to produce N41–68. In our reconstituted transcription system, aberrant stop-start transcription within the *N* gene using these cryptic signals by the cap-defective mutants causes rapid attenuation of transcription, diminishing synthesis of full-length *N* mRNA and downstream mRNAs (Ogino, 2014; Neubauer et al., 2016). In contrast, Li et al. (2008, 2009) showed that the same or similar cap-defective mutations (G1154A, T1157A, H1227A, and R1228A) in the VSV L protein cause premature termination of *N* mRNA synthesis at various sites with a modest preference for U-rich sequences within the *N* gene, but almost randomly, resulting in generation of uncapped (5′-ppp-, 5′-pp-, or 5′-HO-) transcripts of heterogeneous lengths (100 to 500 nt). Furthermore, these mutations were reported to repress 3′-polyadenylation of transcripts when using their reconstituted transcription system in the presence of rabbit reticulocyte lysates (Li et al., 2008, 2009). However, in our system (Ogino, 2014; Neubauer et al., 2016), the cap-defective mutations do not affect 3′-polyadenylation of uncapped full-length *N* mRNA as well as N157–1326, indicating that there is no link between 5′-capping and 3′-polyadenylation. The reasons for these differences are currently unclear. It is interesting to note that, in systems for HRSV (*Pneumoviridae*), putative PRNTase inhibitors as well as mutations in a putative PRNTase domain of the L protein also cause premature termination of mRNA synthesis, resulting in production of 5′-uncapped abortive transcripts (< 50 nt) (Liuzzi et al., 2005; Braun et al., 2017). Thus, it can be suggested that the co-transcriptional L–pRNA intermediate formation and subsequent pRNA transfer (pre-mRNA capping) by the PRNTase domain of the L proteins are essential for further elongation of pre-mRNA into full-length mRNA, and the PRNTase domain serves as a key regulatory domain controlling accurate stop-start transcription.

## Conservation of PRNTase or PRNTase-Like Domains Among NNS RNA Viruses and Their Related Viruses

**FIGURE 12:**
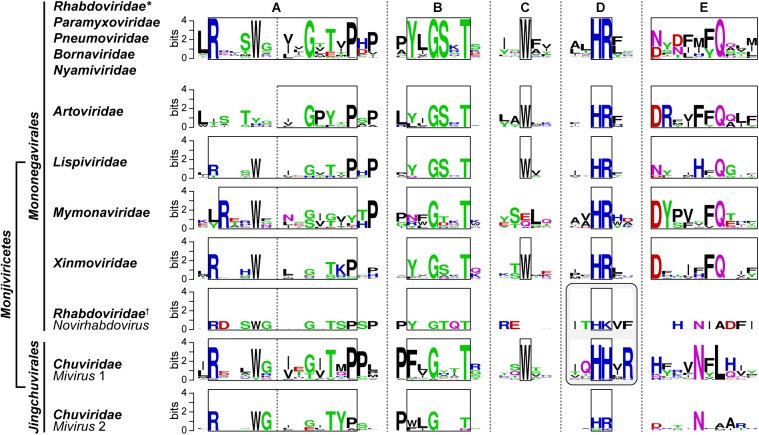
Conservation of PRNTase motifs in L proteins of NNS RNA viruses and their related viruses. Local amino acid sequences (PRNTase motifs A–E) of PRNTase-like domains in L proteins of 7 peropuviruses (*Artoviridae*), 6 arliviruses (*Lispiviridae*), 10 sclerotimonaviruses (*Mymonaviridae*), 8 anpheviruses (*Xinmoviridae*), and 4 novirhabdoviruses (*Rhabdoviridae*^†^) belonging to the order *Mononegavirales* and 29 miviruses [group 1, 23 viruses with a circular genome(s); group 2, 6 viruses with a linear genome, *Chuviridae*] belonging to the order *Jingchuvirales* were analyzed by the WebLogo program (Crooks et al., 2004). The resulting sequence logos are shown with those for NNS RNA viruses belonging to the *Rhabdoviridae* (^∗^, except novirhabdoviruses), *Paramyxoviridae, Filoviridae, Bornaviridae*, and *Nyamiviridae* families (top, as in Figure 8A).

As reported before (Neubauer et al., 2016), L proteins of NNS viruses belonging to the *Rhabdoviridae, Paramyxoviridae, Pneumoviridae* (formerly *Pneumovirinae, Paramyxoviridae*), *Filoviridae, Bornaviridae*, and *Nyamiviridae* families possess a PRNTase or PRNTase-like domain with the conserved motifs A–E (Figure 12, top). Here, we further analyzed amino acid sequences of L proteins of newly discovered NNS RNA viruses belonging to the *Artoviridae, Lispiviridae, Mymonaviridae, Sunviridae*, and *Xinmoviridae* families in the order *Mononegavirales* and related negative strand RNA viruses, miviruses with different types of genome (I, circular non-segmented; II, circular segmented; and III, linear non-segmented) belonging to the *Chuviridae* family in the order *Jingchuvirales* (Li et al., 2015; Shi et al., 2016; Siddell et al., 2019). The *Mononegavirales* and *Jingchuvirales* orders constitute the class *Monjiviricetes* in the *Haploviricotina* subphylum of the phylum *Negarnaviricota* (Siddell et al., 2019). L proteins of all these NNS RNA viruses and their related miviruses were found to possess a PRNTase-like domain with conserved PRNTase motifs or their similar sequences (Figure 12). The L protein of Sunshine Coast virus (formerly Sunshine virus), a sole member of the *Sunviridae* family, also has these motifs (not shown) (see Neubauer et al., 2016). As in the case of novirahbdoviral L proteins (Neubauer et al., 2016), motif C is absent in L proteins of 7 sclerotimonaviruses (*Mymonaviridae*), and 6 miviruses (group 2, genome type III), while motif E is absent in those of all known miviruses. Interestingly, L proteins of 4 novirahbdoviruses (Ogino et al., 2010; Ogino and Banerjee, 2011a; Neubauer et al., 2016) and 22 miviruses (group 1, genome types I and II) have HK and HH sequences, respectively, as candidates for their counterparts of motif D. It is intriguing to note that R1228 in motif D of the VSV PRNTase domain can be functionally replaced with histidine although to a lesser degree, but not with lysine (Ogino et al., 2010), thereby supporting the hypothesis that the HH sequence is miviral counterpart of motif D. One notable exception is the L protein of Shuāngào lacewing virus (mivirus type III, GenBank id: KM817613), in which key catalytic residues in motifs B and D are not conserved.

**FIGURE 13:**
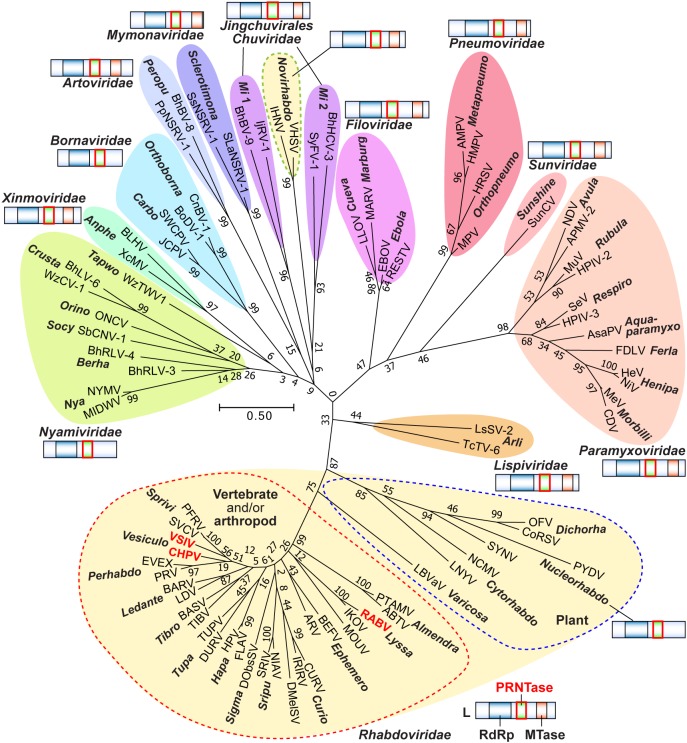
Phylogenetic analysis of PRNTase and PRNTase-like domains in L proteins of NNS RNA viruses and their related viruses. Amino acid sequences of core PRNTase and PRNTase-like domains of selected NNS RNA viral L proteins were aligned using the PSI-Coffee program^3^ (Di Tommaso et al., 2011). A phylogenetic analysis based on the alignment was performed using the Molecular Evolutionary Genetics Analysis (MEGA) software (version 7.0) (Kumar et al., 2016). A phylogenetic tree was generated by the Maximum Likelihood method with 1,000 bootstrap repetitions. Colored branches represent different families. Genus names are placed close to the virus names. The numbers at the nodes indicate bootstrap values. The scale bar shows the branch length corresponding to 0.5 amino acid substitutions per site. The domain organization of L proteins of NNS RNA viruses belonging to the indicated families is schematically represented. Virus names (abbreviations, GenBank accession nos., and amino acid residue ranges) are as follows: vesicular stomatitis Indiana virus (VSIV, K02378, 1081–1302), Chandipura virus (CHPV, AJ810083, 1071–1292), spring viremia of carp virus (SVCV, AJ318079, 1069–1290), pike fry rhabdovirus (PFRV, FJ872827, 1069–1290), perch rhabdovirus (PRV, JX679246, 1093–1310), eel virus European X (EVEX, FN557213, 1070–1287), Le Dantec virus (LDV, KM205006, 1085–1309), Barur virus (BARV, KM204983, 1080–1302), Tibrogargan virus (TIBV, GQ294472, 1098–1321), Bas-Congo virus (BASV, JX297815, 1097–1319), Durham virus (DURV, FJ952155, 1075–1296), tupaia virus (TUPV, AY840978, 1077–1297), Flanders virus (FLAV, AF523199, 1097–1318), Hart Park virus (HPV, KM205011, 1098–1319), Drosophila melanogaster sigmavirus (DMelSV, GQ375258, 1084–1307), Drosophila obscura sigmavirus (DObsSV, GQ410979, 1100–1323), Niakha virus (NIAV, KC585008, 1084–1306), Sripur virus (SRIV, KM205023, 1083–1305), Curionopolis virus (CURV, KJ701190, 1118–1338), Iriri virus (IRIRV, KM204995, 1113–1333), bovine ephemeral fever virus (BEFV, AF234533, 1108–1331), Adelaide River virus (ARV, JN935380, 1107–1329), Moussa virus (MOUV, FJ985748, 1101–1314), rabies virus (RABV, M13215, 1093–1320), Ikoma lyssavirus (IKOV, JX193798, 1093–1320), Puerto Almendras virus (PTAMV, KF534749, 1077–1298), Arboretum virus (ABTV, KC994644, 1077–1298), lettuce big-vein associated virus (LBVaV, AB075039, 1075–1286), lettuce necrotic yellows virus (LNYV, AJ867584, 1064–1294), northern cereal mosaic virus (NCMV, AB030277, 1058–1278), potato yellow dwarf virus (PYDV, GU734660, 1082–1304), sonchus yellow net virus (SYNV, L32603, 1142–1362), orchid fleck virus (OFV, AB244418, 1073–1286), coffee ringspot virus (CoRSV, KF812526, 1073–1276), infectious hematopoietic necrosis virus (IHNV, X89213, 1078–1300), viral hemorrhagic septicemia virus (VHSV, Y18263, 1076–1298), Measles virus (MeV, M20865, 1132–1370), canine distemper virus (CDV, Y09629, 1132–1370), Hendra virus (HeV, AF017149, 1191–1429), Nipah virus (NiV, AF212302, 1191–1429), Fer-de-Lance virus (FDLV, AY141760, 1127–1365), Atlantic salmon paramyxovirus (AsaPV, EF646380, 1135–1374), Sendai virus (SeV, X03614, 1132–1372), human parainfluenza virus 3 (HPIV-3, M21649, 1132–1372), mumps virus (MuV, D10575, 1142–1380), human parainfluenza virus 2 (HPIV-2, X57559, 1140–1378), Newcastle disease virus (NDV, AY262106, 1112–1350), avian paramyxovirus 2 (APMV-2, EU338414, 1144–1382), human respiratory syncytial virus-A2 (HRSV-A2, M75730, 1198–1419), murine pneumonia virus (MPV, AY729016, 1133–1354), avian metapneumovirus (AMPV, U65312, 1122–1343), human metapneumovirus (HMPV, AF371337, 1123–1344), Ebola virus (EBOV, AF086833, 1105–1352), Reston virus (RESTV, AF522874, 1105–1350), Marburg virus (MARV, Z29337, 1108–1377), Lloviu virus (LLOV, JF828358, 1102–1349), Borna disease virus 1 (BoDV-1, AJ311522, 993–1213), canary bornavirus 1 (CnBV-1, KC464471, 993–1213), jungle carpet python virus (JCPV, MF135780, 990–1209), southwest carpet python virus (SWCPV, MF135781, 991–1210), Wenzhou tapeworm virus 1 (WzTWV1, KX884436, 1030–1250), Wçnzhōu crab virus 1 (WzCV-1, KM817644, 985–1205), Beihai rhabdo-like virus 6 (BhLV-6, KX884405, 1009–1229), Orinoco virus (ONCV, KX257488, 1006–1227), soybean cyst nematode virus 1 (SbCNV-1, HM849038, 1035–1283), Bìihai rhabdo-like virus 3 (BhRLV-3, KX884408, 1009–1229), Bìihai rhabdo-like virus 4 (BhRLV-4, KX884406, 1023–1241), Midway virus (MIDWV, FJ554525, 1038–1261), Nyamanini virus (NYMV, FJ554526, 1038–1261). Líshí spider virus 2 (LsSV-2, KM817632, 1167–1404), Tachéng tick virus 6 (TcTV-6, KM817641, 1069–1293), Sunshine Coast virus (SunCV, JN192445, 1119–1346), Sclerotinia sclerotiorum negative-stranded RNA virus 1 (SsNSRV-1, KJ186782, 1078–1292), Soybean leaf-associated negative-stranded RNA virus 1 (SLaNSRV-1, KT598225, 1090–1304), Pteromalus puparum negative-strand RNA virus 1 (PpNSRV-1, KX431032, 1034–1258), Bìihai barnacle virus 8 (BhBV-8, KX884410, 1100–1324), Xînchéng mosquito virus (XcMV, KM817661, 1048–1278), Bolahun virus (BLHV, KX148552, 1046–1277), Bìihai barnacle virus 9 (BhBV-9, KX884409, 1073–1320), Imjin River virus 1 (IjRV-1, KU095839, 1038–1279), Shāyáng fly virus 1 (SyFV-1, KM817598, 1114–1364), and Bìihai hermit crab virus 3 (BhHCV-3, KX884404, 1091–1342).

We generated a phylogenetic tree using an amino acid sequence alignment of core regions (for VSV, residues 1081–1302) in PRNTase and PRNTase-like domains of selected viruses (Figure 13). The phylogenetic analysis clustered homologous PRNTase/PRNTase-like domains into evolutionary related groups, which closely correspond to clades of the virus genera or families, as observed for their RdRp domains (Li et al., 2015; Shi et al., 2016; Wolf et al., 2018). Although the PRNTase motifs show the high conservation with the indicated variations (see Figure 12), other regions have been highly diversified during evolution. It is apparent that novirahbdoviral PRNTase-like domains are significantly different from other rhabdoviral PRNTase domains (Ogino et al., 2010; Ogino and Banerjee, 2011a; Neubauer et al., 2016), but rather exhibit similarities to miviral PRNTase-like domains (Figure 13). Finally, since L proteins of nuclear-replicating NNS RNA viruses belonging to the *Nucleorhabdovirus* genus of *Rhabdoviridae, Bornaviridae*, and *Nyamiviridae* have a PRNTase-like domain, but lack an MTase domain (Figure 13; Ogino and Banerjee, 2011a), we suggest that these viruses may use their own capping system and cellular cap methylation systems to produce the cap 1 structure in host cell nuclei.

## Conclusion and Perspectives

As described in this review article, the numerous biochemical and structural studies on the VSV RNA biosynthesis machinery have led to the remarkable discoveries, provoking paradigm shifts in understanding unique roles of NNS RNA viral proteins in transcription and replication. Those include the finding that the rhabdoviral L proteins catalyze the unique mRNA capping reaction by the unconventional mechanism involving their GTPase and PRNTase activities. The striking differences between host and viral mRNA capping systems emphasize the potential of NNS RNA viral PRNTase domains as attractive targets for developing anti-viral agents.

Since a PRNTase-like domain is present in L proteins of all known NNS RNA viruses as well as their related viruses, we propose that these viruses employ the unconventional capping mechanism. However, the inability to establish efficient *in vitro* transcription or capping systems for other NNS RNA viruses has hampered the progress in our understanding of their precise mechanisms of mRNA capping. Our hypothesis is challenged by studies on some paramyxoviral L proteins that suggest that their C-terminal portions may carry out conventional mRNA capping with their RTPase and GTase activities (Gopinath and Shaila, 2009; Nishio et al., 2011; Singh et al., 2015; Ansari et al., 2019), although there is no evidence that these activities are directly involved in paramyxoviral mRNA capping. Therefore, it would be particularly interesting to solve the mechanisms of mRNA capping used by paramyxoviruses.

Although recent technologies have enabled us to generate and purify recombinant L proteins or their fragments for respective enzymatic assays more easily, reliable data on their enzymatic activities can be obtained only when these recombinant proteins are free from cellular or baculoviral (when a baculovirus expression system is used) enzymes (e.g., phosphatases, nucleases, MTases) and/or other impurities that affect the reactions, and their enzymatic products are unambiguously identified using appropriate methods. It is also very important to demonstrate that these recombinant proteins exhibit enzymatic activities and substrate specificities that are the same as or similar to those of their native forms, if available. The classical approach to perform *in vitro* transcription with purified virus particles or RNPs still have advantages in characterizing virion-associated native enzymatic activities. If NNS RNA viral mRNAs start with 5′-adenosine as in the case of VSV (Abraham et al., 1975a,b; Ogino and Banerjee, 2007), it would be possible to identify origins of phosphate groups forming the 5′-5′ triphosphate bridge in the cap structure (GpppA) on mRNAs by performing *in vitro* transcription with GTP or ATP labeled with ^32^P at different positions (i.e., [α, β, or γ-^32^P]GTP or ATP). These studies would provide definitive evidence to distinguish between unconventional and conventional capping mechanisms. Unfortunately, this approach cannot be applied to 5′-G-started mRNAs of NNS RNA viruses, such as HRSV (Barik, 1993). Needless to say, new *in vitro* RNA capping assays including intermediate formation and nucleotidyl transfer assays for other NNS RNA viruses would be necessary to perform detailed mechanistic studies on mRNA capping.

It is now apparent that rhabdoviral PRNTase domains perform covalent catalysis in the pRNA transfer reaction to GDP to produce the cap structure (Ogino and Banerjee, 2007; Ogino et al., 2010). However, the mechanisms underlying the specific recognition of pppAAC-RNA and GDP have not been fully addressed. Structural studies on complexes of the PRNTase domain of the VSV L protein with its substrates, including the covalent L–pRNA intermediate, would certainly gain deeper insights into the roles of amino acid residues in the PRNTase motifs and other sites in substrate recognition and catalysis. Currently, the mechanisms of the cap 1 formation by a transcribing VSV L protein remain largely unknown. Thus, it would be important to understand how the enzymatic domains of the VSV L protein coordinately carry out respective steps of RNA synthesis and processing during the dynamic transcription cycle.

Non-segmented negative strand viral L proteins may have retained evolutionary conserved elements critical for common functions, but diversified some other regions for virus-specific functions. Thus, the important studies would be to explore virus-specific functions of respective NNS RNA viral L proteins. Since mRNAs of NNS RNA viruses belonging to different families are unique due to virus-specific mRNA-start sequences that are distinct from those of rhabdoviruses (Stillman and Whitt, 1997; Kolakofsky et al., 1998), it would be interesting to define how NNS RNA viral PRNTase and MTase domains specifically recognize their own pre-mRNAs for capping and methylation, respectively. On the other hand, the priming-capping loop identified in the VSV and RABV L proteins is conserved among rhabdoviruses infecting vertebrate and arthropod hosts, but not other NNS RNA viruses, at the amino acid sequence level. Thus, it would be curious to elucidate the mechanisms of terminal *de novo* initiation by L proteins of other NNS RNA viruses and identify their priming elements, if any. Detailed investigation along these lines would certainly advance our understanding on how mononegaviral RNA biosynthesis machineries play common and virus-specific roles in transcription and replication at the molecular level, and eventually reveal an Achilles’ heel for a target in developing anti-viral agents.

## Author Contributions

TO conceived and wrote the manuscript, and prepared the figures. TG wrote the part of the manuscript, performed the docking study, and prepared the structural images.

## Conflict of Interest Statement

The authors declare that the research was conducted in the absence of any commercial or financial relationships that could be construed as a potential conflict of interest.

## Footnotes

1 www.modelarchive.org

2 http://weblogo.berkeley.edu/

3 http://tcoffee.crg.cat/
